# Proteolytic activation of fatty acid synthase signals pan-stress resolution

**DOI:** 10.1038/s42255-023-00939-z

**Published:** 2024-01-02

**Authors:** Hai Wei, Yi M. Weaver, Chendong Yang, Yuan Zhang, Guoli Hu, Courtney M. Karner, Matthew Sieber, Ralph J. DeBerardinis, Benjamin P. Weaver

**Affiliations:** 1 Department of Pharmacology, UT Southwestern, Dallas, TX USA; 2 Children’s Medical Center Research Institute, UT Southwestern, Dallas, TX USA; 3 Department of Internal Medicine, UT Southwestern, Dallas, TX USA; 4 Department of Physiology, UT Southwestern, Dallas, TX USA; 5 Howard Hughes Medical Institute, UT Southwestern, Dallas, TX USA

**Keywords:** Homeostasis, Proteolysis, Gene expression, Stress signalling, Metabolism

## Abstract

Chronic stress and inflammation are both outcomes and major drivers of many human diseases. Sustained responsiveness despite mitigation suggests a failure to sense resolution of the stressor. Here we show that a proteolytic cleavage event of fatty acid synthase (FASN) activates a global cue for stress resolution in *Caenorhabditis* *elegans*. FASN is well established for biosynthesis of the fatty acid palmitate. Our results demonstrate FASN promoting an anti-inflammatory profile apart from palmitate synthesis. Redox-dependent proteolysis of limited amounts of FASN by caspase activates a C-terminal fragment sufficient to downregulate multiple aspects of stress responsiveness, including gene expression, metabolic programs and lipid droplets. The FASN C-terminal fragment signals stress resolution in a cell non-autonomous manner. Consistent with these findings, FASN processing is also seen in well-fed but not fasted male mouse liver. As downregulation of stress responses is critical to health, our findings provide a potential pathway to control diverse aspects of stress responses.

## Abstract (web summary)

Wei et al. show that proteolytic cleavage of fatty acid synthase (FASN) upon stress contributes to stress resolution. This role in stress resolution of the resulting C-terminal fragment of FASN is independent of its canonical function in fatty acid synthesis.

## Main

Alterations in nutrient availability, oxidative imbalances and exposures to cytotoxic substances or pollutants are all examples of cellular stressors. When faced with these insults, cells mount the relevant stress mitigation program to restore homeostasis. Outcomes include upregulating buffering factors such as chaperones, repairing damaged DNA, autophagy of cellular components or death of irreversibly damaged cells. Whether animals have a globally acting mechanism to detect the resolution of diverse stressors and signal the downregulation of multiple features of stress responses is unknown; however, such a mechanism would offer a survival advantage to limit loss of energy stores, speed wound healing and prepare the animal for encounters with another stressor. For multicellular organisms, coordinating stress recovery across tissues imposes additional challenges.

Adaptations to stressors not only require altered gene expression but also metabolic rewiring to repurpose energy stores. Metabolic adaptations to stressors can result in the generation of reactive oxygen species (ROS) or reactive nitrogen species. These reactive species cause extensive cellular damage including lipid peroxidation resulting in toxic aldehyde intermediates that further damage other macromolecules and lead to the death of tissues. Thus, lipids have an intriguing duality in stress responses as both energy stores and liability to damaging agents resulting in lipotoxicity. This underscores the need to have dynamic control of lipid availability but also safeguard the cell.

Cytosolic FASN is the biosynthetic enzyme responsible for the de novo synthesis of saturated fatty acids, the building blocks of fats and higher-order lipids^1,2^. Fat synthesis by FASN is a major pathway consuming energy stores in the form of NADPH. Notably, upregulation of *FASN* expression is a hallmark of tumorigenesis where tumor cells are thought to acquire de novo lipid synthesis^3^; however, it is not clear whether *FASN* upregulation solely reflects increased nutrient demand for growth as lipids are involved in multiple aspects of cellular transformation, including immune suppression and drug resistance, thereby preventing death of transformed cells^3^.

Caspases are well known for activating proinflammatory cytokines or committing cells to programmed cell death if a genotoxic insult is not repaired. This same family of cysteine proteases also has ancient nonlethal roles in promoting differentiation; however, across metazoans, these same proteases have poorly understood roles in supporting homeostasis, where modulating caspase function alters stress resistance in *C.* *elegans*^4,5^, reprograms cardiac hypertrophic responses in rats^6^ and accelerates aging in mice^7^. Our previous work identified CED-3 caspase blocking a p38 MAPK-dependent epidermal pathogen response^5^. Also, the dual-leucine zipper kinase (DLK-1 and MAPK3K12) pathway was implicated downstream of caspase in neuronal regeneration following injury^8^. We and others recently showed that the relative extent of caspase activity can be neuroprotective by supporting organelle dynamics where caspase can limit TDP43 mitochondrial injury^9^ or modulate p38 MAPK signaling to support lysosome formation^10^. Additionally, stress-induced caspase-2 cleavage of site 1 protease (S1P) triggers persistent activation of the transcription factor SREBP leading to NASH development^11^; however, for each of these cases, the caspase was found targeting a factor activating a stress response.

Beyond detecting an insult to activate a stress response, little is known how animals sense the resolution of stressors. Here we identify a signaling function of FASN that is independent of palmitate biosynthesis. We show that redox-dependent caspase cleavage of FASN serves as a sensing mechanism to gauge appropriate responses to diverse stressors. With cysteine active sites, the proteolytic activity of caspases can be inhibited by stressors in a redox-dependent manner. When the stressor is mitigated, limited amounts of the C-terminal fragment (CTF) act as a strong cue to downregulate widespread features of animal stress responses, analogous to an anti-inflammatory signal. Moreover, FASN-CTF enzymatic activity is required to suppress stress-responsive gene expression programs and promote lipid mobilization. Forced expression of FASN-CTF before stress amelioration compromises survival underscoring its function as a stress-resolution cue.

## Results

### Caspase controls diverse stress responses

**Fig. 1:**
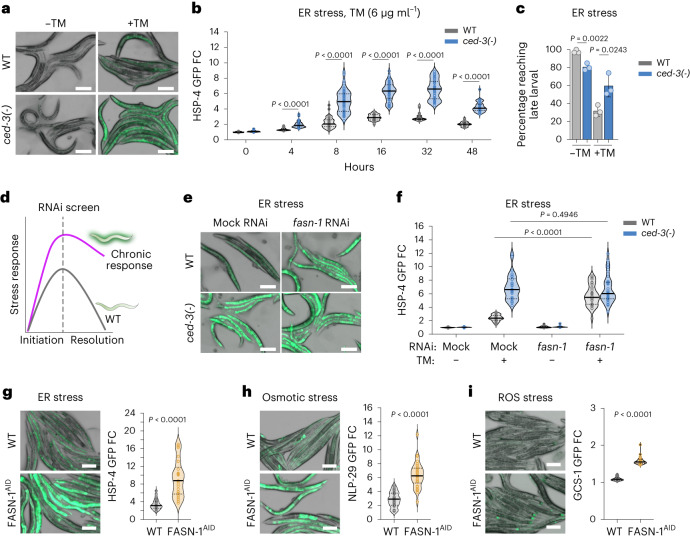
Caspase and FASN limit chronic pan-stress responses. **a**,**b**, Expression (**a**) and quantification (**b**) of intestinal ER stress reporter HSP-4p::GFP (Hsp70 member 5) with tunicamycin (TM) treatment to induce ER stress. WT, wild type. **c**, Quantification of animals escaping larval arrest with TM treatment. **d**–**f**, Targeted RNAi screen for chronic stress response factors (**d**) reveals that *fasn-1* acts like *ced-3* to limit stress response in HSP-4p::GFP expression (**e**) and quantification (**f**). **g**–**i**, Eliminating FASN-1 protein using AID also induces expression of stress reporters under ER (**g**), osmotic (**h**) and ROS (**i**) stresses by TM, sodium chloride and paraquat. Scale bar, 200 μm (**a**,**e**,**g**–**i**). Each circle represents one animal (**b**,**f–i**). Mean pixel intensity of each animal was normalized to mean value of WT at 0 h (no stress) and plotted as fold change (FC). Violin plots show median (solid line) with quartiles (dashed line). For **b**, 8 h, *n* = 50; 16, 32 and 48 h, *n* = 40. Mean ± s.d., *n* = 3 biological replicates (**c**). *n* = 30 animals for each time point (**f**–**i**). *P* values were calculated using a two-tailed Mann–Whitney *U*-test (**b**,**f**–**i**) and unpaired two-tailed *t*-test (**c**). Source data

**Extended Data Fig. 1:**
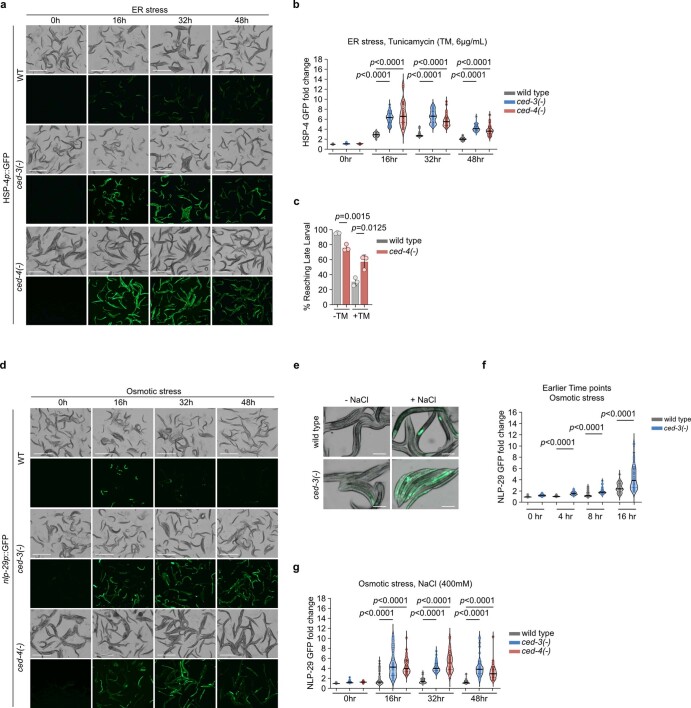
Elevated ER and osmotic stress responses in *ced-3(-)* and *ced-4(-)* animals. **a-b**, representative images (**a**) and quantification (**b**) of HSP-4p::GFP stress reporter induction under ER stress at different time points. Wild type and *ced-3(-)* were repeated from main figure for comparison to *ced-4(-)* animals. n = 40 animals. **c**, quantification of *ced-4(-)* animals escaping larval arrest with tunicamycin (TM) treatment to induce ER stress. n = 3 biological replicates. mean ± SD. **d**, representative images of NLP-29p::GFP (antimicrobial peptide) stress reporter induction with 400 mM NaCl treatment to induce osmotic stress in *ced-3(-)* and *ced-4(-)* animals at different time points. **e**, digital zoom in of 0 h and 32 h images shown in **d** of epidermal NLP-29p::GFP reporter induction in *ced-3(-)* animals under osmotic stress. **f**, quantification of NLP-29p::GFP stress reporter expression under osmotic stress at early time points. n = 50 animals. **g**, quantification of NLP-29p::GFP stress reporter expression for both *ced-3(-)* and *ced-4(-)* animals. n = 40 animals. For **a,d**, scale bar, 1 mm. For **e**, scale bar, 200μm. For **b,f,g** each circle represents one animal. Mean pixel intensity of each animal was normalized to mean value of wild type at 0 hr (no stress) and plotted as fold change. Violin plots show median (solid line) with quartiles (dashed line). *p* values were calculated using Mann-Whitney, two-tailed test (**b**,**f**,**g**) and unpaired two-tailed t-test (**c**). Source data

Disrupting the caspase pathway in *C.* *elegans* provided us with a model to examine chronic stress responses. As early as 4 h of tunicamycin treatment, the *ced-3(-)* caspase and upstream activating Apaf gene *ced-4(-)* null mutants had dramatically enhanced induction of the Hsp70 family (HSPA5, BiP) endoplasmic reticulum (ER) stress marker HSP-4p::GFP relative to wild-type animals (Fig. 1a,b and Extended Data Fig. 1a,b). This enhanced response persisted to 2 d (Fig. 1b). Moreover, although the *ced-3(-)* and *ced-4(-)* null mutants normally develop slower than wild-type animals, both mutants had faster development under ER stress conditions compared to wild-type animals (Fig. 1c and Extended Data Fig. 1c), suggesting that the higher induction of HSP-4p::GFP reflects an enhanced survival advantage upon tunicamycin-induced ER stress.

**Extended Data Fig. 2:**
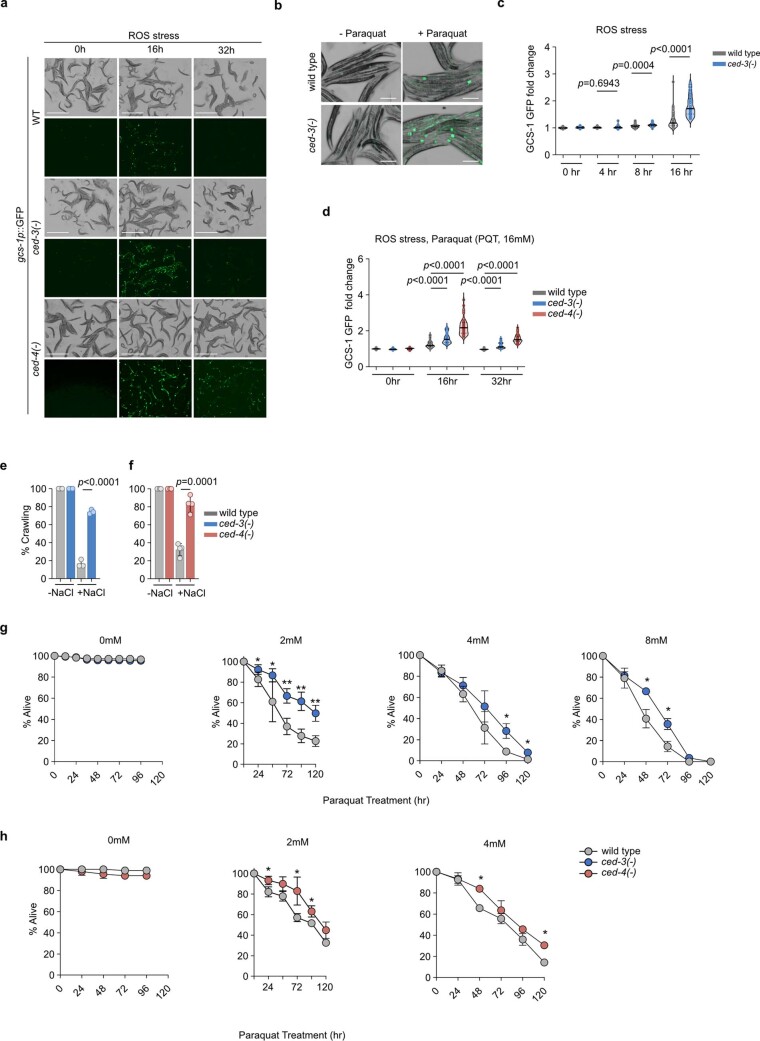
Elevated ROS stress response in *ced-3(-)* and *ced-4(-)* animals. **a**, representative images of GCS-1p::GFP (glutamate-cysteine ligase) stress reporter induction with 16 mM paraquat (PQT) treatment to induce ROS stress in *ced-3(-)* and *ced-4(-)* animals at different time points. scale bar, 1 mm. **b**, digital zoom in of 0 h and 16 h of neuronal and intestinal GCS-1p::GFP reporter induction in *ced-3(-)* animals under ROS stress. scale bar, 200μm. **c**, quantification of GCS-1p::GFP stress reporter induction under ROS stress at early time points. n = 50 animals. **d**, quantification of GCS-1p::GFP stress reporter induction for both *ced-3(-)* and *ced-4(-)* animals. n = 40 animals. **e-f**, quantification of motile animals for *ced-3(-)* (**e**) and *ced-4(-)* (**f**) mutants with sodium chloride (NaCl) treatment to induce osmotic stress. *ced-3(-)* n = 3 and *ced-4(-)* n = 4 biological replicates. Mean ± SD. **g-h**, survival for *ced-3(-)* (**g**) and *ced-4(-)* (**h**) mutants with increasing concentration of PQT to induce ROS stress. n = 3 biological replicates. Mean ± SD, total animals examined in **g**, 0 mM, n = 532; 2 mM, n = 579, 4 mM, n = 493, 8 mM, n = 567; total animals examined in **h**, 0 mM, n = 505; 2 mM, n = 567, 4 mM, n = 286. *, *p* < 0.05; **, *p* < 0.0001. Exact *p* value see Source Data. For **c,d**, each circle represents one animal. Mean pixel intensity of each animal was normalized to mean value of wild type at 0 hr (no stress) and plotted as fold change. Violin plots show median (solid line) with quartiles (dashed line). *p* values were calculated using Mann-Whitney, two-tailed test (**c,d**) and unpaired two-tailed t-test (**e,f,g,h**). Source data

We next sought to test whether *ced-3* and *ced-4* mutants would have similar persistent phenotypes with other stressors. Analogous to the heightened ER stress response, the *ced-3(-)* and *ced-4(-)* null mutants had dramatically enhanced induction of markers for both osmotic stress (NLP-29p::GFP) and ROS stress (GCS-1p::GFP) relative to wild-type animals when challenged with high salt and paraquat, respectively (Extended Data Figs. 1d–g and 2a–d).

The *ced-3(-)* and *ced-4(-)* mutants also had enhanced motility when given the salt challenge (Extended Data Fig. 2e,f) and enhanced survival on paraquat relative to wild-type animals (Extended Data Fig. 2g,h). Our findings are consistent with previous work showing that *ced-3* and *ced-4* mutants have enhanced survival with ER and osmotic stresses^4^. Notably, for each of the diverse stress challenges, wild-type animals never reached the same pronounced induction as seen with the *ced-3* caspase and *ced-4* Apaf mutants. Also, the *ced-3* and *ced-4* mutants had marked variability for the induction of the stress reporters. Together, these results indicate that caspase pathway normally regulates the overall magnitude of responsiveness to diverse stressors. We were therefore intrigued to identify what target pathway is responsible for the enhanced pan-stress responsiveness.

### FASN acts like caspase to limit stress responses

**Extended Data Fig. 3:**
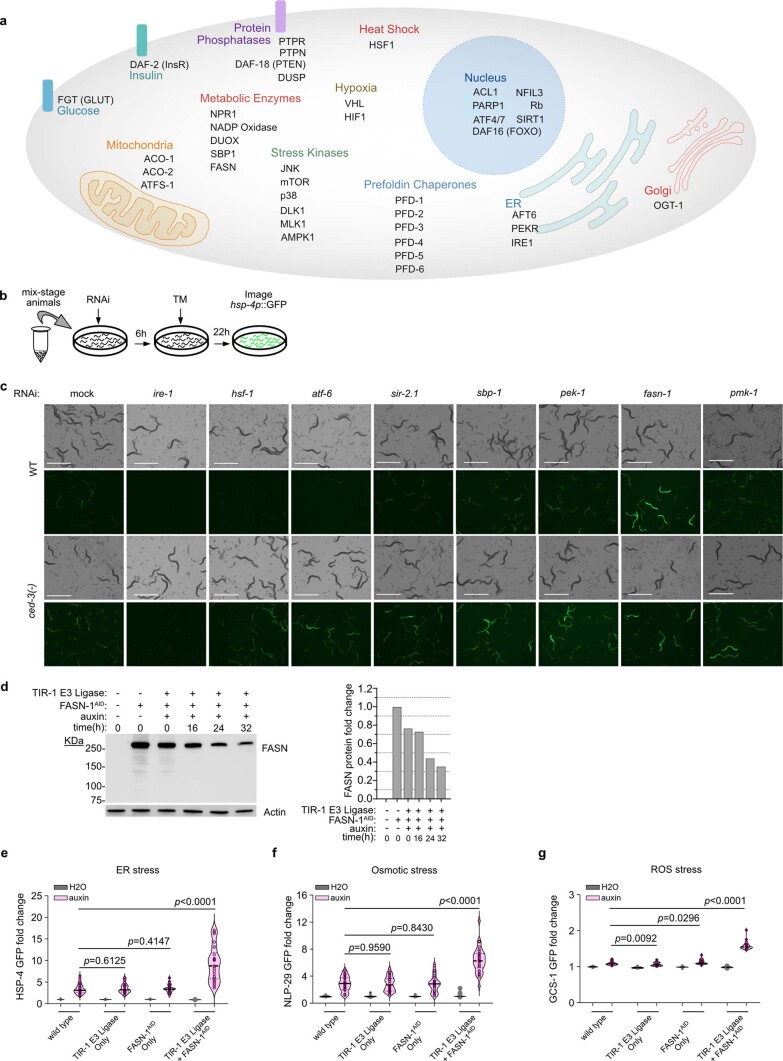
Target RNAi screen to reveal factors modulating heightened stress responses and characterization of auxin-induced degradation of FASN-1. **a**, diagram showing all factors in multiple stress response pathways included in the targeted RNAi screen. **b**, work flow of RNAi screen. **c**, representative images of raw images from RNAi screen. Scale bar, 1 mm. Screen was performed once with duplicates. Candidates were later confirmed with one additional round of confirmation screen. **d**, optimization for time-dependent auxin-induced degradation of FASN. Optimization was performed once to show timing of degradation. **e**-**g**, HSP-4p:: GFP (**e**), NLP-29p:: GFP (**f**) and GCS-1p:: GFP (**g**) signals are not affected by expressing TIR-1 E3 ligase transgene or insertion of auxin induced degron at the endogenous locus of *fasn-1*. Wild type auxin treated and TIR-1 E3 Ligase + FASN^AID^ auxin treated conditions were repeats from main figures for comparison. n = 30 animals for each condition. *p* values were calculated using Mann-Whitney, two-tailed test. Source data

To reveal the caspase signaling target, we performed an RNA interference (RNAi) screen using the HSP-4p::GFP reporter with tunicamycin-induced ER stress to identify candidate factors contributing to persistently-heightened stress responses (Fig. 1d). The targeted screen examined established stress-responsive regulators and genes upregulated in stress (Extended Data Fig. 3a–c). The *C.* *elegans* FASN ortholog *fasn-1* gene acted like *ced-3(-)* and *ced-4(-)* mutants with enhanced induction and pronounced variability of HSP-4p::GFP expression under ER stress (Fig. 1e,f and Extended Data Fig. 3c). Moreover, because *fasn-1* RNAi knockdown did not enhance stress response in *ced-3(-)* mutants (Fig. 1f), we wanted to confirm that loss of FASN-1 expression acts like *ced-3(-)* null.

To confirm that the loss of FASN-1 function resulted in enhanced stress response in animals with intact CED-3 function, we used an alternative technique to deplete FASN at the protein level. We added hemagglutinin (HA)-labelled auxin-induced degron (AID) tags to both the N and C termini of the *fasn-1*-coding sequence in the endogenous genomic locus using CRISPR mutagenesis. When treated with auxin, the amount of FASN protein was significantly reduced (Extended Data Fig. 3d) and depletion enhanced induction of HSP-4p::GFP with ER stress (Fig. 1g), NLP-29p::GFP for osmotic stress (Fig. 1h) and GCS-1p::GFP for ROS stress (Fig. 1i). These effects were not due to TIR-1 E3 ligase expression, AID-tagging of FASN-1 or auxin treatment as these alone did not enhance the reporters (Extended Data Fig. 3e–g). Because the loss of *fasn-1* acted like the loss of *ced-3* caspase, we considered the possibility that FASN-1 could be acting downstream of CED-3 in limiting stress responses.

### FASN proteolysis by caspase limits stress response

**Fig. 2:**
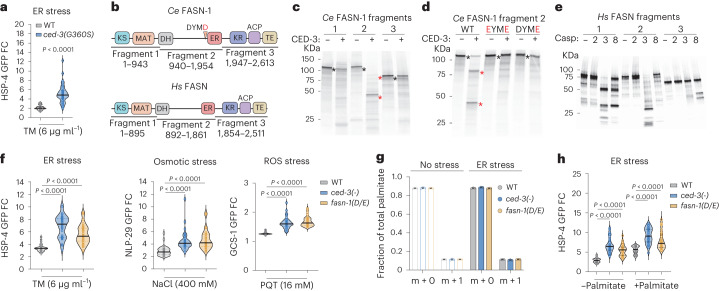
FASN activated by caspase cleavage limits pan-stress responses independent of palmitate synthesis. **a**, Expression of HSP-4p::GFP by caspase active-site mutation G360S. **b**, Highly conserved FASN domains and fragments used for in vitro caspase cleavage analyses. Enzymatic domains indicated with square boxes. *Ce*, *C.* *elegans*; *Hs*, *H.* *sapiens*. **c**,**d**, CED-3 in vitro cleavage of ^35^S-labelled *C.* *elegans* FASN-1 fragments (**c**), the DYMD to DYME mutation (D/E) in fragment 2 blocks CED-3 cleavage (**d**). Red asterisks indicate cleaved products; black asterisks indicate full-length molecules. **e**, Conserved cleavage. ^35^S-labelled human FASN is also cleaved in fragment 2 by caspase-3 producing a stable CTF similar to *C.* *elegans*. Casp, caspase-2, caspase-3 and caspase-8 as indicated for each lane. **f**, Cleavage-resistant *fasn-1(D/E) C.* *elegans* mutant phenocopies *ced-3(-)* mutant for enhanced inductions of ER, Osmotic and ROS stress reporters. **g**, Measurement of palmitate synthesis using 50% deuterated water (D_2_O) labelling for 16 h in the presence or absence of ER stress. m + 0, unlabelled palmitate; m + 1, deuterium-labelled palmitate representing newly synthesized palmitate. *n* = 3 biological replicates for each. Mean ± s.d. **h**, HSP-4p::GFP expression under ER stress with palmitate supplementation. Each circle represents one animal (**a**,**f**,**h**). Mean pixel intensity of each animal was normalized to mean value of WT without stress and plotted as FC. Violin plots show median (solid line) with quartiles (dashed line). For **a**, *n* = 40 animals for each genotype. *n* = 30 animals for each condition (**f**,**h**). *P* values were calculated using a two-tailed Mann–Whitney *U*-test. Cleavage assays (**c**–**e**) were repeated twice independently with similar results. Source data

**Extended Data Fig. 4:**
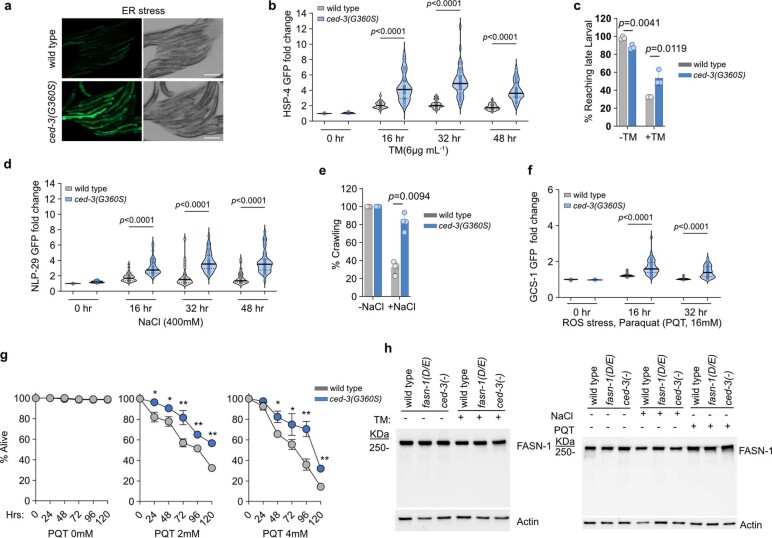
Elevated ER, osmotic and ROS stress responses in *ced-3(G360S)* active site mutant animals and *in vivo* expression of cleavage resistant *fasn-1(D/E)* protein. **a**, representative images of HSP-4p::GFP stress reporter induction under ER stress in *ced-3(G360S)* animals. scale bar, 200μm. **b**, quantification of HSP-4::GFP stress reporter induction in *ced-3(G360S)* animals under ER stress at different time points. n = 40 animals. Treatment at 32 hr was repeated from main figure (Fig. 2a) for comparison. **c**, quantification of *ced-3(G360S)* animals escaping larval arrest with tunicamycin (TM) treatment to induce ER stress. n = 3 biological replicates. mean ± SD. **d**, quantification of NLP-29p::GFP stress reporter induction in *ced-3(G360S)* animals under osmotic stress at different time points. n = 40 animals. **e**, quantification of motility *ced-3(G360S)* mutants under osmotic stress. n = 4 biological replicates. mean ± SD. **f**, quantification of GCS-1p::GFP stress reporter induction in *ced-3(G360S)* animals under ROS stress at different time points. n = 40 animals. **g**, survival for *ced-3(G360S)* animals with increasing concentration of PQT to induce ROS stress. n = 3 biological replicates. Mean ± SD, total animals examined, 0 mM, n = 218; 2 mM, n = 354, 4 mM, n = 286. *, *p* < 0.05. **, *p* < 0.01. Exact *p* value see Source Data. **h**, Western blot analyses of endogenous FASN-1 tagged with HA-AID tag in the absence of TIR-1 Ligase to allow expression under no stress, ER stress (TM), osmotic (NaCl) and ROS (PQT) stress in wild type, *ced-3(-)* and *fasn-1(D/E)* mutant animals. Western blot was performed once to shown no change in expression. For **b,d,f**, each circle represents one animal. Mean pixel intensity of each animal was normalized to mean value of wild type at 0 hr (no stress) and plotted as fold change. Violin plots show median (solid line) with quartiles (dashed line). *p* values were calculated using two-tailed Mann-Whitney test (**b,d,f**) and unpaired, two-tailed t-test (**c,e,g**). Source data

To confirm that the proteolytic activity of caspase is required for limiting stress responses, we used a proteolytic dead caspase active-site mutation G360S. This mutant generates a full-length CED-3 protein but lacks proteolytic activity. Consistent with *ced-3* null mutants, animals with proteolytic dead CED-3 also had enhanced stress reporter induction as well as survival under ER, osmotic and ROS stressors (Fig. 2a and Extended Data Fig. 4a–g). This finding suggests that the proteolytic activity of CED-3 is required for regulating stress response. It also explains the role of CED-4 Apaf in stress response as it promotes CED-3 proteolytic activity.

To test directly the cleavage of FASN-1 by CED-3, we divided *C.* *elegans* FASN-1 into three large peptide fragments and tested each with in vitro cleavage analyses (Fig. 2b). We identified a single CED-3 cleavage site for *C.* *elegans* FASN-1 in fragment 2 at Asp1593 toward the end of the long non-enzymatic linker region separating the N- and C-terminal enzymatic domains (Fig. 2c,d). Like all animal FASN proteins, the human FASN protein has the same domain architecture as *C.* *elegans* FASN-1 with long intervening non-enzymatic linker. Therefore, we similarly divided *Homo* *sapiens* FASN into three large peptide fragments. We found that human caspase-3 cleaved human FASN fragments 1 and 2 in vitro (Fig. 2e). This cleavage leaves an intact CTF, including most of the enoyl reductase (ER) domain and ketoacyl reductase (KR), acyl carrier protein (ACP) and thioesterase (TE) domains analogous to the cleavage location for *C.* *elegans* FASN-1. Furthermore, human caspase-3 and *C.* *elegans* CED-3 caspase are known to have similar proteolytic activities. Altogether these results indicate that the cleavage of FASN by caspase may be broadly conserved in metazoans.

To test whether the cleavage of FASN-1 by CED-3 is responsible for the enhanced stress responsiveness in vivo, we generated a FASN-1 cleavage-resistant mutation D1593E ‘*fasn-1(D/E*)’ in the endogenous locus of *C.* *elegans fasn-1* using CRISPR mutagenesis. Using western blot, we first tested FASN-1 expression in wild-type, *ced-3(-)* and *fasn-1(D/E*) mutants. Notably, we did not observe any quantitative accumulation of FASN-1 cleavage fragments in *C.* *elegans* in the absence or presence of stressors (Extended Data Fig. 4h). These results suggest the possibility that only a small fraction of this highly abundant protein is cleaved by CED-3 in vivo.

**Extended Data Fig. 5:**
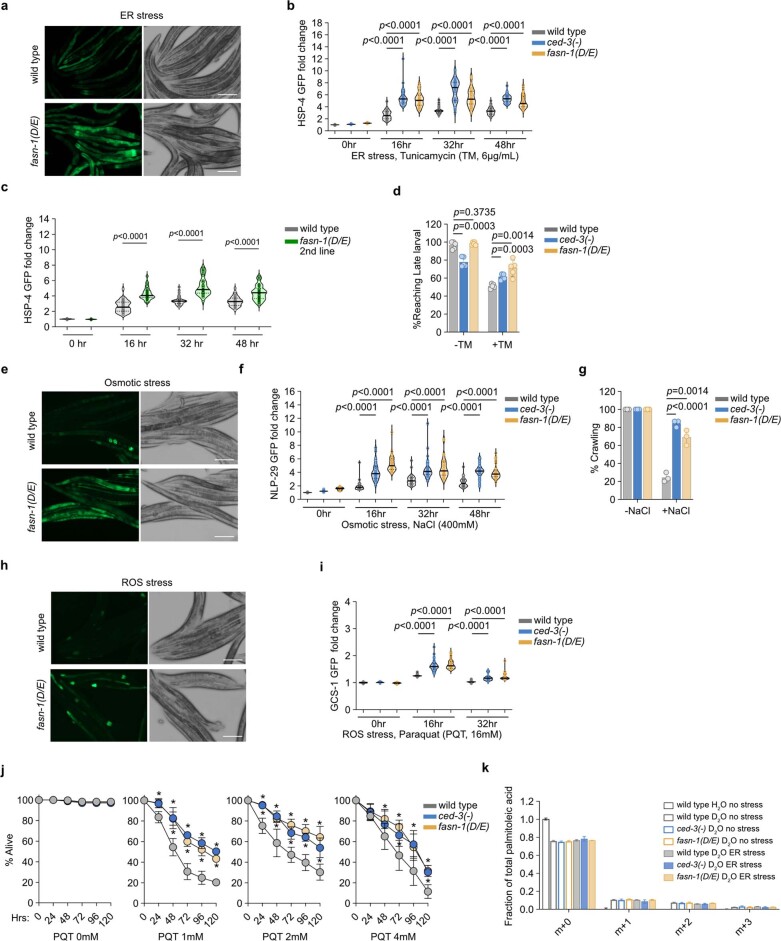
Elevated ER, osmotic and ROS stress responses in cleavage-resistant *fasn-1(D/E)* mutant animals. **a**, representative images of HSP-4p::GFP stress reporter induction under ER stress in *fasn-1(D/E)* animals. scale bar, 200μm. **b**, quantification of HSP-4::GFP stress reporter induction in *fasn-1(D/E)* animals under ER stress at different time points. Treatment at 32 hr was repeated from main figure (Fig. 2f) for comparison. n = 30 animals. **c**, quantification of HSP-4::GFP stress reporter induction in a second CRISPR line of *fasn-1(D/E)* animals under ER stress at different time points. n = 30 animals. **d**, quantification of *fasn-1(D/E)* animals escaping larval arrest with tunicamycin (TM) treatment to induce ER stress. n = 5 biological replicates. mean ± SD. **e**, representative images of NLP-29p::GFP stress reporter induction under osmotic stress in *fasn-1(D/E)* animals. scale bar, 200μm. **f**, quantification of NLP-29p::GFP stress reporter induction in *fasn-1(D/E)* animals under osmotic stress at different time points. Treatment at 32 hr was repeated from main figure (Fig. 2f) for comparison. n = 30 animals. **g**, quantification of *fasn-1(D/E)* animals motility with NaCl treatment to induce osmotic stress. n = 3 biological replicates. mean ± SD. **h**, representative images of GCS-1p::GFP stress reporter induction under ROS stress in *fasn-1(D/E)* animals. scale bar, 200μm. **i**, quantification of GCS-1p::GFP stress reporter induction in *fasn-1(D/E)* animals under ROS stress at different time points. Treatment at 16 hr was repeated from main figure (Fig. 2f) for comparison. n = 30 animals. **j**, survival for *fasn-1(D/E)* animals with increasing concentration of PQT to induce ROS stress. n = 5 biological replicates. Mean ± SD, total animals examined, 0 mM, n = 593; 1 mM, n = 604; 2 mM, n = 621; 4 mM, n = 536. *, *p* < 0.05. Exact *p* value see Source Data. **k**, measurement of palmitoleic acid synthesis using 50% deuterated water (D_2_O) labeling for 16 hours in the presence or absence of ER stress. m + 0, unlabeled palmitoleic acid. m + 1, m + 2, m + 3 are deuterium-labeled representing newly synthesized palmitoleic acid. n = 3 biological samples. Mean ± SD. *p* values were calculated using Mann-Whitney test (**b,f,i**) and unpaired t-test (**d,g,j**). Source data

We then tested the impact of *fasn-1(D/E)* on stress response. Similar to the *ced-3(-)* null mutant, the *fasn-1(D/E)* mutant had upregulated HSP-4p::GFP in response to tunicamycin (ER stress, Fig. 2f). This enhanced upregulation was equivalent to *ced-3(-)* (Extended Data Fig. 5a,b) and was confirmed with an independent second *fasn-1(D/E)* CRISPR mutant (Extended Data Fig. 5c). The *fasn-1(D/E)* mutant had enhanced survival comparable to *ced-3(-)* mutants (Extended Data Fig. 5d).

We next sought to test whether the *fasn-1(D/E)* mutant would have similar persistent phenotypes with other stressors. Analogous to the heightened ER stress response, the *fasn-1(D/E)* had dramatically enhanced induction of markers for both osmotic stress (NLP-29p::GFP) and ROS stress (GCS-1p::GFP) equivalent to *ced-3(-)* when challenged with high salt and paraquat, respectively (osmotic and ROS stresses; Fig. 2f and Extended Data Fig. 5e–j).

Altogether, these data suggest that CED-3 cleaves FASN-1 at the Asp1593 residue to limit pan-stress responsiveness in vivo. Because failure of cleavage caused by the *fasn-1(D/E)* mutation phenocopied the loss of caspase activity seen with the *ced-3(-)* mutant, we concluded that a low level of caspase cleavage activates a distinct function of FASN to limit a pan-stress response.

### FASN cleavage does not impact palmitate synthesis

The saturated fatty acid palmitate (C16) is the major end product of all metazoan FASN enzymes^2^. To test whether *ced-3(-)* and *fasn-1(D/E)* mutations have compromised palmitate synthesis, we performed metabolic labelling with D_2_O to monitor fatty acid synthesis. We found no significant alterations in palmitate synthesis (Fig. 2g) or palmitoleate synthesis (Extended Data Fig. 5k) for *ced-3(-)* or *fasn-1(D/E)* mutants with or without ER stress.

To further confirm that the enhanced stress responsiveness of *ced-3(-)* and *fasn-1(D/E)* mutants was not due to a fatty acid deficiency, we supplemented animals with palmitate under ER stress and found that palmitate supplementation did not reduce the heightened HSP-4p::GFP induction in *ced-3(-)* null animals or *fasn-1(D/E)* cleavage-resistant mutants (Fig. 2h). In contrast, extra palmitate enhanced HSP-4p::GFP induction in wild-type, *ced-3(-)* and *fasn-1(D/E)* animals (Fig. 2h). Altogether, these results indicate that the stress-resolution function of cleaved FASN is independent of palmitate synthesis.

### FASN cleavage by caspase impacts redox metabolism

**Fig. 3:**
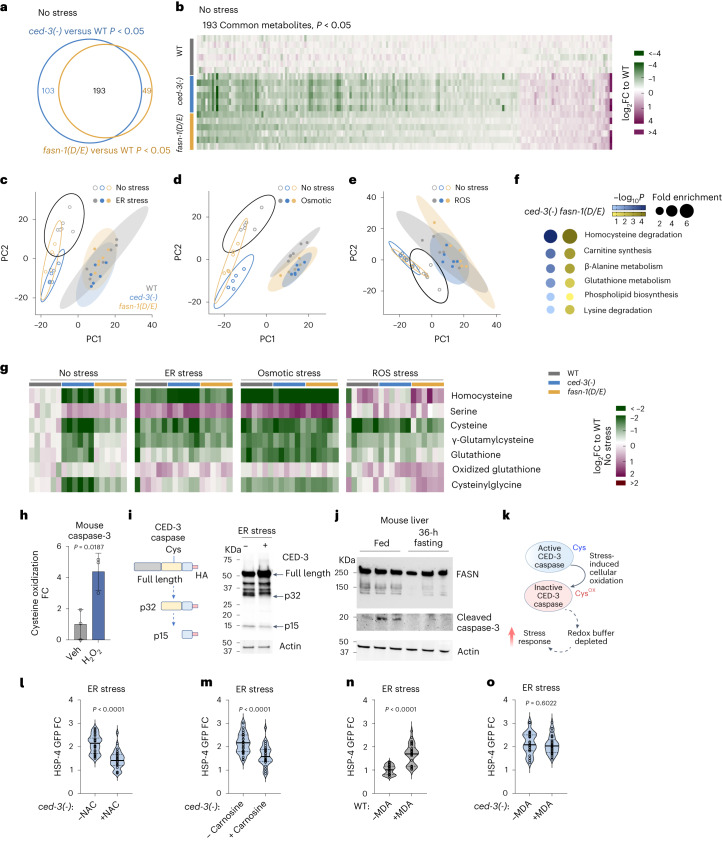
Interplay of cellular redox state and FASN cleavage determines stress responsiveness. **a**,**b**, Alterations of metabolites in *ced-3(-)* and *fasn-1(D/E)* animals compared to WT in the absence of stress from untargeted metabolomics. Untargeted metabolomics with *P* < 0.05 as cutoff revealed 193 overlapping metabolites (**a**) shown in heat map (**b**) in *ced-3(-)* and *fasn-1(D/E)*. *n* = 6 biological replicates. **c**–**e**, PCA of untargeted metabolites for WT, *ced-3(-)* and *fasn-1(D/E)* animals with ER (**c**), osmotic (**d**) and ROS (**e**) stressors. **f**, Metabolite enrichment analyses for *ced-3(-)* null and *fasn-1(D/E)* mutants compared to WT using MetaboAnalyst with the Small Molecule Pathway Database (SMPDB). False discovery rate (FDR) < 0.1. Input list from metabolites shown in **a** with *P* < 0.05, *ced-3(-)* 296 and *fasn-1(D/E)* 242 metabolites. **g**, Heat map of detected metabolites in glutathione pathway. log_2_FC relative to mean value of WT no stress. **a**–**g**, *n* = 6 biological replicates. *P* values calculated using an unpaired two-tailed *t*-test (**a**,**b**,**g**) or one-tailed Fisher’s exact test (**f**). **h**, Caspase-3 cysteine oxidation in primary mouse calvarial osteoblasts (cOBs) using DCP-Bio1 IP MS. Average abundance of caspase-3-oxidized cysteine treated with vehicle was set to 1.0. Total caspase-3 protein in lysate (Extended Data Fig. 6f). Veh, water. *n* = 3 biological replicates. Mean ± s.d. **i**, CED-3 auto-processing monitored by western blot of endogenous CED-3 with a C-terminal HA tag under ER stress. CED-3 auto-processing with ER stress was repeated independently twice with similar results. **j**, Western blot of liver extracts from well-fed or 36-h-fasted male mice showing processing of FASN and cleaved caspase-3. *n* = 3 male mice for each condition. **k**, Diagram of caspase cys active site as a function of oxidative state and impact on stress responsiveness. **l**,**m**, Expression of HSP-4p::GFP under ER stress with NAC (**l**) or carnosine (**m**) antioxidant supplementation in *ced-3(-)* animals. **n**,**o**, Induction of HSP-4p::GFP under ER stress with oxidized lipid metabolite MDA supplementation in WT (**n**) and *ced-3(-)* animals (**o**). Each circle represents one animal. Mean pixel intensity of each animal normalized to mean value of WT without stress and plotted as FC. Violin plots show median (solid line) with quartiles (dashed line). *P* values were calculated using an unpaired, two-tailed *t*-test. *n* = 3 biological replicates (**h**) or two-tailed Mann–Whitney *U*-test. *n* = 30 animals for each time point (**l**–**o**). Source data

**Extended Data Fig. 6:**
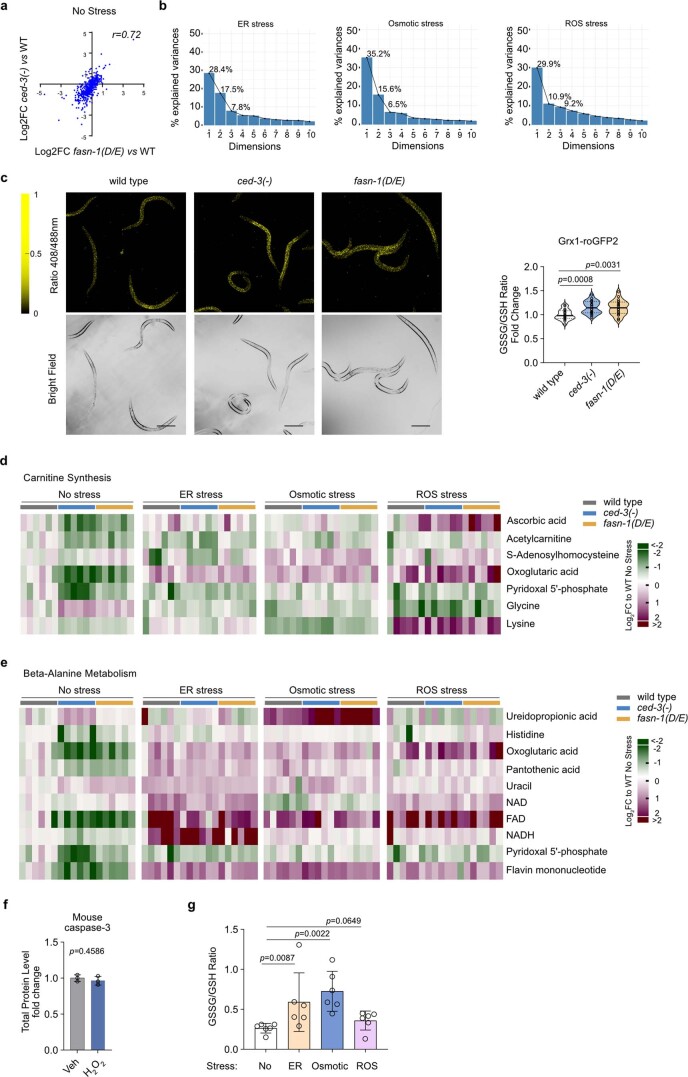
Effect of *ced-3(-)* and *fasn-1(D/E)* mutation on metabolism with diverse stressors. **a**, Pearson’s analysis of metabolites changed in *fasn-1(D/E)* and *ced-3(-)* null mutants compared to wild type animals with no stress treatment. **b**, eigenvalues of PCA analysis for wild type, *ced-3(-)* and *fasn-1(D/E)* mutant with no stress treatment and each of the ER, osmotic and ROS stress. **c**, images and quantification of Grx1-roGFP2 ratio in wild type, *ced-3(-)* and *fasn-1(D/E)* under no stress condition. Excitation of 408 nm was used to estimate GSSG and excitation of 488 nm was used to estimate GSH. Ratio-metric images of the two channels was generated using Fiji (Image J v 1.54 f). Scale bar, 200μm. WT n = 17, *ced-3(-)* and *fasn-1(D/E)* n = 20. **d-e**, heatmap of detected metabolites in carnitine synthesis (**d**) and beta-alanine metabolism (**e**) pathway. Log_2_-fold change relative to mean value of wild type no stress. n = 6 biological replicates. **f**, total caspase-3 protein detected in lysate. Average of vehicle treated set to 1, n = 3. Mean ± SD. **g**, GSSG/GSH ratio of wild type animals under diverse stress conditions. n = 6 biological replicates. Mean ± SD. Data from untargeted metabolomics. *p* values were calculated using Mann-Whitney, two-tailed test (**c,g**) or unpaired, two-tailed t-test (**f**). Source data

To reveal the metabolic impact of CED-3 caspase on FASN-1, we used untargeted metabolomics in *C.* *elegans* to analyse the profiles of wild-type, *ced-3(-)* and *fasn-1(D/E)* strains in normal and stress conditions (Supplementary Data 1). When comparing *ced-3(-)* to wild-type, we found that 296 metabolites were significantly altered in the absence of stress (Fig. 3a). Additionally, when comparing *fasn-1(D/E)* to wild-type, we found that 242 metabolites were significantly altered (Fig. 3a). Of the 193 metabolites commonly altered in both mutants, 191 were changed in the same direction (Fig. 3b). Using Pearson’s analysis, we also showed a strong positive correlation between *ced-3(-)* and *fasn-1(D/E)* (Extended Data Fig. 6a). Thus, *fasn-1(D/E)* shows 65% of the metabolic changes seen in *ced-3(-)* mutant before stress, reflecting a substantial common subset of metabolic alterations in both mutants.

To understand the variation for all detected metabolites, we used principal component analysis (PCA). Most of the variability was well represented in the first two principal components for each of the treatment conditions (Extended Data Fig. 6b). We found that the biggest variance in metabolites (PC1) corresponded to stress treatments, including ER (Fig. 3c), osmotic (Fig. 3d) and ROS stressors (Fig. 3e). The second largest variance represented by the PC2 axes corresponded to the different genotypes in the absence of stress. PC2 showed that the *ced-3(-)* and *fasn-1(D/E)* mutants clustered closer to each other (open circles, Fig. 3c–e), suggesting that the metabolic profiles of these two mutants are more similar to each other compared to the wild type. Notably, the dispersion among the genotypes on PC2 axes collapsed upon stress treatment (closed circles, PC2 axes; Fig. 3c–e). The clustering of wild-type with the mutants upon stress treatments suggests a subset of metabolites already altered in *ced-3(-)* and *fasn-1(D/E)* that normally occurs during the stress response.

Using enrichment analysis of metabolites altered in *ced-3(-)* or *fasn-1(D/E)* mutants, we found a significant enrichment for redox adaptations for both mutants in the absence of stress (Fig. 3f). Most homocysteine and glutathione metabolites detected are depleted in both *ced-3(-)* and *fasn-1(D/E)* mutants in the absence of stress (no stress; Fig. 3g). Of note, the same depletion pattern was observed for wild-type animals under ER and osmotic stress conditions. ROS stress had a somewhat different pattern compared to the other two stressors but still resulted in an overall reduction of the cysteine pool (Fig. 3g). The glutathione pathway modulates cellular redox to mitigate stress responses. Unlike other stressors, ROS stress directly impacts cellular redox and paraquat detoxification directly modulates this pathway.

To further verify the alteration in redox metabolism, we used the Grx1-roGFP2 construct previously established to reflect the ratio of oxidized to reduced glutathione^12,13^. We found that *ced-3(-)* and *fasn-1(D/E)* mutants both have an increased ratio of oxidized to reduced glutathione in the absence of stress compared to wild-type (Extended Data Fig. 6c).

We also analysed other enriched pathways for *ced-3(-)* and *fasn-1(D/E)*, including carnitine and β-alanine pathways, and found that *ced-3(-)* and *fasn-1(D/E)* both showed most metabolites in those pathways decreased before stress but these alterations did not reflect the changes seen during stress (Extended Data Fig. 6d,e). Altogether, the metabolomic analyses revealed that both *ced-3(-)* null and the *fasn-1(D/E)* mutants had significant alterations in redox metabolism that resembles a stress-responsive state before encounter of a stressor.

### Cell redox modulates caspase activity in stress

As a cys protease, caspases can be inhibited by oxidizing species^14^ and highly oxidizing environments can even suppress caspase-mediated apoptosis^15,16^. To assess the relative alteration in caspase cysteine oxidation in cells as a result of exposure to oxidative stress, we treated primary mouse osteoblasts with hydrogen peroxide overnight. To enrich oxidized cysteine residues, we used the DCP-Bio1 probe that forms adducts with singly-oxidized cysteines (cysteine sulfenic acid). We observed more than a threefold increase in oxidized caspase-3 cysteine residues when treated with the oxidizing agent hydrogen peroxide compared to vehicle-treated cells (Fig. 3h) without affecting caspase-3 protein levels (Extended Data Fig. 6f). These findings suggest that caspase cysteine residues are sensitive to cellular redox conditions. We next considered whether oxidized cellular environments caused by other stressors would impact caspase activity.

ER stress both results from the accumulation of (and itself generates) ROS^17,18^. To test whether cellular oxidation is induced by ER stress, we examined the ratio of oxidized to reduced glutathione metabolites and observed an increased ratio in oxidized to reduced glutathione in *C.* *elegans* (Extended Data Fig. 6g), suggesting a more-oxidative cellular environment under ER stress. Because caspase auto-processing requires enzymatic activity of caspase, we tested how ER stress affects CED-3 auto-processing in *C.* *elegans*. We found that overnight ER stress treatment resulted in diminished CED-3 auto-processing in vivo (Fig. 3i). ER stress increased the accumulation of full-length CED-3 protein that corresponded to decreased accumulations of both the p32 and p15 processed subunits (Fig. 3i). This observation indicates that CED-3 has diminished proteolytic capability under ER stress.

In mammals, liver is a tissue enriched for FASN expression^19,20^ and fasting induces oxidative stress on the liver^21,22^. Therefore, we analysed the effect of fasting on FASN and caspase expression in male mouse liver. During the well-fed state, we found FASN processing along with the presence of active caspase-3, whereas both were absent during prolonged fasting (Fig. 3j). Altogether, these findings indicate that caspase cysteine oxidation and subsequent inhibition of caspase activity can occur during stress conditions. The decrease of caspase activity would thereby lead to further depletion of GSH redox buffer causing a heightened stress response similar to the *ced-3(-)* mutation (Fig. 3k).

**Extended Data Fig. 7:**
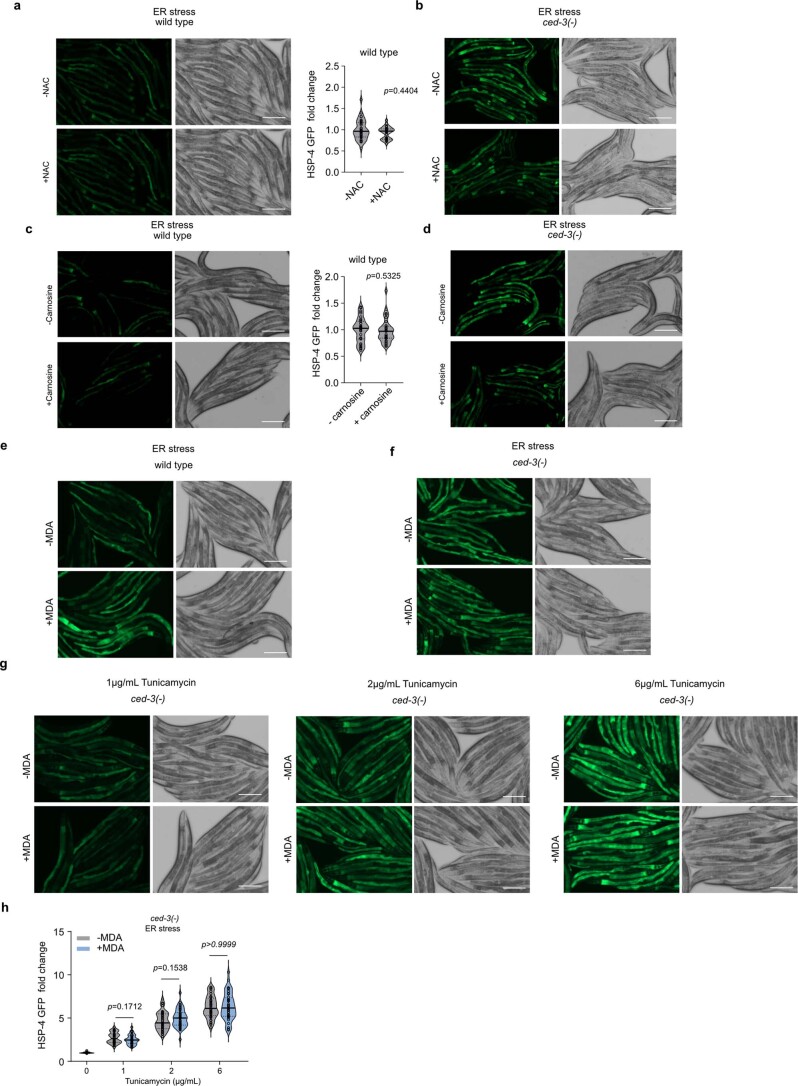
Extended Data Fig.7 Cellular oxidative state alters caspase-mediated stress responsiveness. **a**, representative images of HSP-4p::GFP stress reporter induction under ER stress with N-acetylcysteine (NAC) supplementation in wild type animals. Quantification n = 30 animals. **b**, representative images of HSP-4p::GFP stress reporter induction under ER stress with NAC supplementation in *ced-3(-)* animals. Quantification shown in main Fig. 3. **c**, representative images of HSP-4p::GFP stress reporter induction under ER stress with carnosine supplementation in wild type animals. Quantification n = 30 animals. **d**, representative images of HSP-4p::GFP stress reporter induction under ER stress with carnosine supplementation in *ced-3(-)* animals. Quantification shown in main Fig. 3. **e-f**, representative images of HSP-4p::GFP stress reporter induction under ER stress with MDA (malondialdehyde) supplementation in wild type animals (**e**) and *ced-3(-)* animals (**f**). Quantification shown in main Fig. 3. **g-h**, representative images (**g**) and quantification (**h**) of HSP-4p::GFP stress reporter induction under increasing tunicamycin concentration with MDA (malondialdehyde) supplementation in *ced-3(-)* animals. n = 40 animals. *p* values were calculated using Mann-Whitney, two-tailed test (**a,c,h**). Scale bar, 200μm (**a**,**b**,**c**,**d**,**e**,**f**,**g**). Source data

To further test whether modulating cellular redox status directly impacts CED-3 caspase-dependent stress responsiveness, we supplemented *ced-3(-)* animals with antioxidants *N*-acetylcysteine (NAC; Fig. 3l and Extended Data Fig. 7a,b) or l-carnosine (Fig. 3m and Extended Data Fig. 7c,d) under ER stress conditions. We found that both antioxidant supplements significantly reduced the heightened ER stress response in *ced-3(-)* mutants. These findings suggest that the CED-3 caspase-mediated stress response can be reversed by increasing the cellular reductive capacity.

Conversely, we then considered how organismal stress response would be impacted by shifting the redox state toward an oxidative profile. Lipid peroxidation occurs as a result of oxidative bursts and elevated cellular ROS results in accumulation of lipid-derived reactive aldehydes such as malondialdehyde (MDA). When supplementing animals with MDA, we found that wild-type animals had increased response to ER stress similar to *ced-3(-)* mutants without MDA (Fig. 3n and Extended Data Fig. 7e,f). Moreover, MDA did not further increase the elevated response of *ced-3(-)* mutants (Fig. 3o). To eliminate the possibility of reaching the reporter detection limit, we repeated the same assay at lower concentrations of tunicamycin and found that the ER stress reporter in *ced-3(-)* mutants was not further enhanced by MDA even at low TM concentrations well below maximal responsiveness (Extended Data Fig. 7g,h). This result indicates that elevated cellular oxidative environment augments stress response and loss of CED-3 caspase mimics an elevated oxidative cellular environment. Because *ced-3(-)* null and the cleavage-resistant *fasn-1(D/E)* mutants both had increased amplitude in stress responses, we concluded that CED-3 proteolytic activity may be redox sensitive to modulate the magnitude of response, suggesting the possibility of FASN cleavage as a signal to scale down stress response.

### FASN-CTF attenuates pan-stress response

**Fig. 4:**
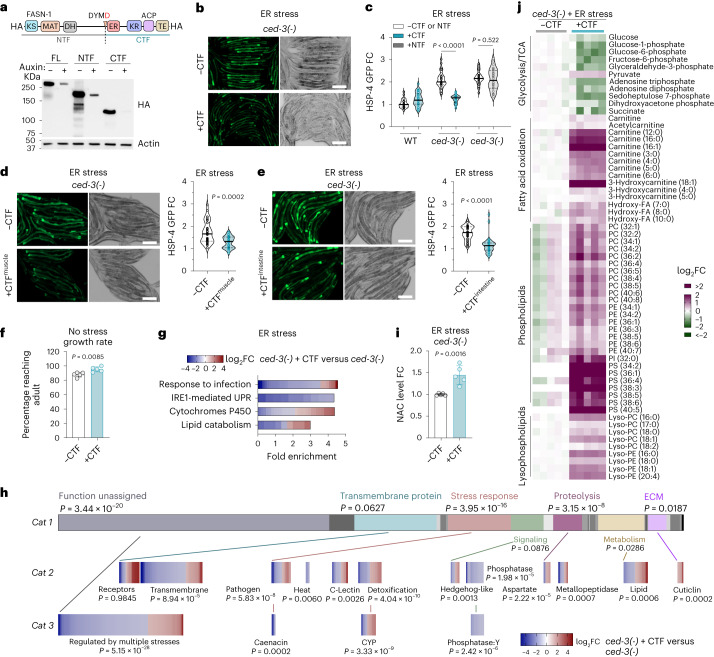
FASN-CTF diminishes chronically elevated stress-responsive gene expression and metabolic programs. **a**, Western blot showing in vivo stabilities of FASN-NTF and CTF. Auxin-controlled endogenous FASN-1 (FL) or a single-copy transgene expressing either NTF or CTF all contain an HA tag for detection. Western blot for stability of FASN fragments was repeated twice independently with similar results. **b**,**c**, HSP-4p::GFP expression (**b**) and quantification (**c**) under ER stress with expression of FASN-CTF or NTF in *ced-3(-)*. *n* = 30 animals. **d**,**e**, HSP-4p::GFP induction under ER stress in *ced-3(-)* animals with expression of FASN-CTF only in muscle (**d**) or only in intestine (**e**). *n* = 30 animals (**d**,**e**). Scale bar, 200 μm (**b**,**d**,**e**). **f**, Growth rate of animals with and without expression of FASN-CTF. *n* = 5 biological replicates with total of *n* = 441 (−CTF) and *n* = 303 (+CTF) animals were assayed. Mean ± s.d. **g**,**h**, Impact of FASN-CTF on *ced-3*(-) mutant for alterations in gene expression under ER stress using the gene set analysis tool in WormBase (wormbase.org/tools/enrichment/tea/tea.cgi) (**g**) and WormCat (wormcat.com/) (**h**). Heat maps (**g**,**h**) show mRNA log_2_FC of *ced-3(-)* mutant expressing CTF compared to *ced-3(-)* with no CTF expression. *n* = 3 biological replicates. FDR < 0.1 and absolute value of log_2_FC > 1 were thresholds for enrichment analysis (**g**,**h**). **i**, NAC level in *ced-3(-)* with and without CTF expression. *n* = 5 biological replicates. Mean ± s.d. **j**, Impact of FASN-CTF expression on *ced-3(-)* mutant for selected metabolites under ER stress. Heat map shows log_2_FC of *ced-3(-)* mutant expressing CTF compared to *ced-3(-)* with no CTF expression. *n* = 5 biological replicates. FA, fatty acid; PC, phosphatidylcholine; PE, phosphatidylethanolamine; PI, phosphatidylinositol; PS, phosphatidylserine; Lyso-PC, lysophosphatidylcholine; Lyso-PE, lysophosphatidylethanolamine; TCA, tricarboxylic acid. *P* values were calculated using an unpaired two-tailed *t*-test (**f**,**i**), two-tailed Mann–Whitney *U*-test (**c**–**e**) or one-tailed Fisher’s exact test (**h**). Source data

**Extended Data Fig. 8:**
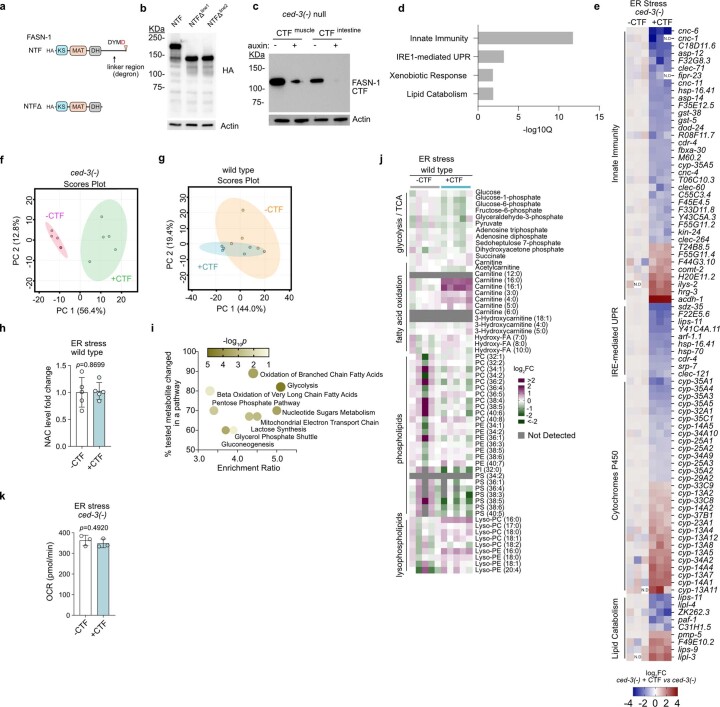
Impact of FASN-CTF on gene expression and metabolic profiles. **a**, diagram showing the location of FASN-NTF linker region which is a degron. NTFΔ is FASN-NTF with the linker region removed. **b**, Western blot of FASN-NTF showing the band near 150 kDa corresponds to removal of the linker region (NTFΔ).Western blot was performed once to confirm FASN-NTF linker region is a degron as shown in Fig.4a. **c**, Western blot of tissue specific CTF expression controlled by auxin. Western blot was performed once to show expression. **d**, FASN-CTF alters gene expression of *ced-3(-)* null mutants pathway enrichments –log_10_Q value. Enrichment analysis was performed using Enrichment Analysis tool from WormBase (https://wormbase.org/tools/enrichment/tea/tea.cgi). **e**, Heatmap of genes in enriched pathways with FDR < 0.1. n = 3 biological replicates. Log_2_ fold change of *ced-3(-)* expressing CTF compared to *ced-3(-)* with no CTF. **f**, PCA analysis for targeted metabolomics of *ced-3(-)* expressing CTF (green) compared to *ced-3(-)* with no CTF (purple). **g**, PCA analysis for targeted metabolomics of wild type animals expressing CTF (green) compared to wild type with no CTF (orange). **h**, N-acetylcysteine (NAC) level in wild type animals with and without CTF expression. n = 5 biological replicates. Mean ± SD. **i**, Enrichment analysis of targeted metabolomics of *ced-3(-)* expressing CTF compared to *ced-3(-)* with no CTF using MetaboAnalyst (https://www.metaboanalyst.ca/) with SMPDB. **j**, heatmap of wild type animals expressing CTF under ER stress showing Log_2_FC of the same metabolites shown in Fig. 4j. n = 5 biological replicates. **k**, OCR analysis of *ced-3(-)* mutant expressing CTF compared to *ced-3(-)* with no CTF expression. n = 3 biological replicates. Mean ± SD. *p* values were calculated using unpaired, two-tailed t-test (**h,k**) or one-tailed Fisher’s exact test (**i**). Source data

To understand the functional outcome of CED-3 cleavage on FASN in vivo, we used CRISPR mutagenesis to insert either the N- or C-terminal cleaved fragment as a single extra copy in *C.* *elegans* (Fig. 4a). The FASN N-terminal fragment (NTF) generated a spectrum of processing bands, whereas the CTF made one stable product (Fig. 4a). Further analysis of the FASN-NTF revealed that the linker region at the end of the NTF likely contains a degron when exposed by proteolytic cleavage (Extended Data Fig. 8a,b).

To analyse the impact of both FASN NTFs and CTFs in stress resolution, we tested the effects of expressing either fragment in *ced-3(-)* null mutants upon stress (Fig. 4b,c). We found that the expression of FASN-CTF is sufficient to resolve the enhanced stress responsiveness of the *ced-3(-)* mutant, whereas the NTF is not (Fig. 4b,c). Moreover, tissue-specific expression of FASN-CTF in either the intestine or muscle (Extended Data Fig. 8c) also decreased the heightened induction of the intestinal HSP-4p::GFP of the *ced-3(-)* mutant (Fig. 4d,e). Therefore, we concluded that FASN-CTF can function cell non-autonomously to decrease the stress response.

Because FASN is an essential gene for de novo fatty acid synthesis and loss of FASN-1 causes developmental arrest in *C.* *elegans*^5,23^, we wanted to determine whether the FASN-CTF works in a dominant negative manner to inhibit full-length FASN de novo fatty acid synthesis. As loss of FASN function compromises development, we measured the growth rate of animals with and without CTF expression to assess the impact on development. We found that animals expressing FASN-CTF had no growth delay compared to animals not expressing the CTF (Fig. 4f). These findings suggest that the FASN-CTF functions to decrease stress response without compromising essential de novo fatty acid synthesis function of FASN during development.

### FASN-CTF promotes anti-inflammatory profile

To further investigate the impact of FASN-CTF on gene expression, we performed mRNA-seq analysis (Supplementary Data 2). We found that with ER stress, expressing the CTF in *ced-3(-)* decreased expression of genes enriched for pathogen defence and IRE1-mediated unfolded protein response (UPR) (Fig. 4g and Extended Data Fig. 8d,e). We also saw mixed alterations in genes enriched for cytochrome P450 and lipid metabolism. Genes in pathogen defence and UPR were almost all downregulated by FASN-CTF (Fig. 4g and Extended Data Fig. 8d,e). Finding suppression of immune and stress-responsive gene expression suggests a strong anti-inflammatory-type function for the FASN-CTF.

To overcome limitations of BLAST-based Gene Ontology enrichment, we also used the WormCat gene set enrichment analysis tool to analyse these genes in more detail^24^. WormCat also identified pathogen response, heat stress, cellular detoxification and lipid metabolism as highly enriched (Fig. 4h). In addition, the new analysis identified an enrichment of a large set of genes with no functional assignment but regulated by multiple stresses (Fig. 4h). More than 70% of these genes were downregulated with the expression of FASN-CTF. Moreover, WormCat analysis revealed new enrichment for transmembrane proteins, proteolysis factors and tyrosine phosphatase, suggesting a possible role of FASN-CTF in cellular remodeling and signaling.

We also analysed the metabolic impact of expressing FASN-CTF on *ced-3(-)* and wild-type animals under ER stress using targeted metabolomics (Supplementary Data 3 and Extended Data Fig. 8f,g). Of note, we found elevated NAC antioxidant with FASN-CTF expression in *ced-3(-)* under ER stress but not in wild-type animals (Fig. 4i and Extended Data Fig. 8h). This finding was consistent with our observation that supplementing NAC reduces the heightened ER stress response in *ced-3(-)* null mutants but not wild-type animals (Fig. 3l and Extended Data Fig. 7a).

We also found that *ced-3(-)* null mutants with ectopic expression of FASN-CTF had altered enrichments for metabolites in multiple energy production pathways (Extended Data Fig. 8i). Specifically, when expressing FASN-CTF in *ced-3(-)* null mutants, of metabolites tested, most of the glycolysis and tricarboxylic acid cycle pathway metabolites were decreased (Fig. 4j). Conversely, almost all the carnitine species, hydroxy-fatty acids and phospholipids tested were increased with FASN-CTF expression (Fig. 4j), suggesting the possibility of altered fatty acid oxidation. With intact CED-3 function in wild-type animals, the FASN-CTF had a much less-pronounced impact on the metabolic profile with a mild increase in carnitine metabolites (Extended Data Fig. 8j), suggesting that the CTF-mediated metabolic changes suppress the lack of CED-3 function.

To address the possibility of altered respiration, we tested the impact of FASN-CTF on oxygen consumption rate (OCR) but did not observe any obvious alteration of *ced-3(-)* with or without CTF expression (Extended Data Fig. 8k), suggesting that substrate switching rather than altered metabolic rates may be more predominate. Altogether, we found that expressing FASN-CTF attenuates stress-responsive gene programs, elevates reducing metabolites and, notably, shifts metabolic substrate composition with elevated soluble lipids.

### FASN-CTF resolves stress-induced lipid droplets

**Fig. 5:**
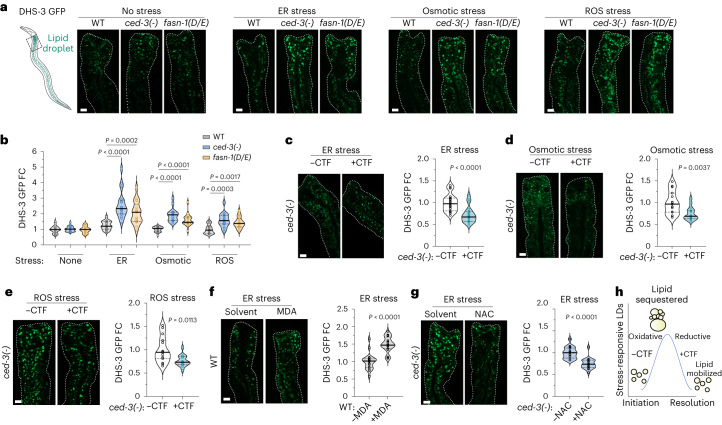
FASN-CTF resolves stress-induced lipid droplets. **a**,**b**, Lipid droplets visualized (**a**) and quantified (**b**) in the same region of intestine by DHS-3::GFP marker intensity under ER, osmotic and ROS stresses. *n* = 15 for osmotic and ROS, *n* = 16 for no treatment and ER stress except *fasn-1(D/E)* *n* = 17 animals per condition. **c**–**e**, Lipid droplets with expression of FASN-CTF in *ced-3(-)* mutant animals under ER stress, *n* = 19 animals (**c**), osmotic stress, *n* = 15 animals (**d**) and ROS stress, *n* = 15 animals (**e**). **f**,**g**, Lipid droplets with supplementation of MDA in WT animals (**f**) and NAC in *ced-3(-)* animals (**g**) under ER stress. *n* = 19 animals on control and 20 animals on MDA. *n* = 17 animals on control and 19 animals on NAC. **h**, Diagram illustrating relationship of lipid droplet (LD) accumulation with CTF expression and cellular oxidative status. Scale bar, 10 μm (**a**,**c**–**g**). *P* values were calculated using a two-tailed Mann–Whitney *U*-test (**b**–**g**). Source data

**Extended Data Fig. 9:**
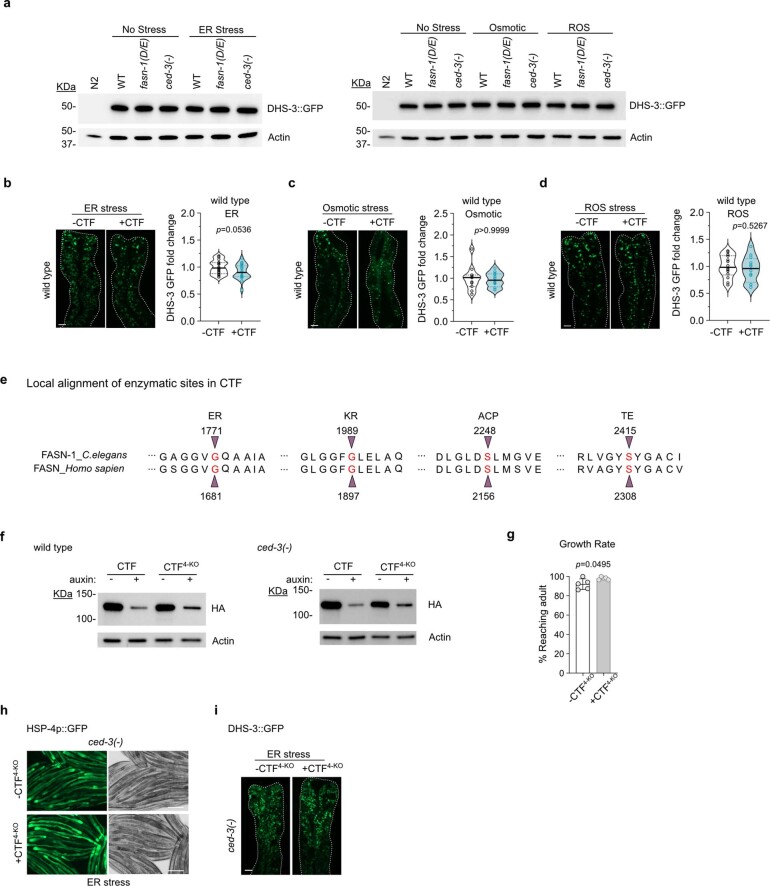
Impact of FASN-CTF on lipid droplet dynamics and characterization of FASN-CTF^4-KO^. **a**, Western blot analysis showing no effect on DHS-3::GFP protein accumulation by *ced-3(-)* or *fasn-1(D/E)* mutants with or without diverse stressors. Western blot was performed once to show no difference in expression. **b-d**, lipid droplets with expression of FASN-CTF in wild type animals under ER stress (**b**), n = 19 animals with CTF and 20 animals without CTF; osmotic stress (**c**), n = 12 animals with CTF and 11 animals without CTF and ROS stress (**d**), n = 12 animals with CTF and 14 animals without CTF. **e**, local sequence alignment of FASN-CTF with four point mutations that inactivate the four enzymatic domains (FASN-CTF^4-KO^). **f**, Western blot showing auxin-controlled expression of FASN-CTF and FASN-CTF^4-KO^ in wild type and *ced-3(-)* animals. Western blot was performed once to show no difference in expression. **g**, no delay in growth rate with expression of FASN-CTF^4-KO^. Percent of animals reaching young adulthood 62 hours after synchronized egg laying. n = 5 biological replicates. Mean ± SD. **h**, representative image of induction of ER stress marker HSP-4p::GFP with expression of FASN-CTF^4-KO^ under ER stress in *ced-3(-)* mutant animals. Quantification shown in main Fig. 6b. **i**, representative image induction of lipid droplet marker DHS-3p::GFP with expression of FASN-CTF^4-KO^ under ER stress in *ced-3(-)* mutant animals. Quantification shown in main Fig. 6c. *p* values were calculated using Mann-Whitney, two-tailed test (**b,c,d**) or unpaired, two-tailed t-test (**g**). Scale bar, 10μm (**b**,**c**,**d**,**i**) and 200μm (**h**). Source data

Lipid droplets serve as readouts of stress responses as they play essential roles in mitigating stress responses, integrating lipid metabolism with energy homeostasis, supporting proteostasis during stress responses, sequestering reactive oxidizing species and supporting immune signaling^25–27^. To observe intestine-specific lipid droplets, we used the DHS-3::GFP fusion protein as a previously established marker in *C.* *elegans*^28^. Without stress treatment, *ced-3(-)* null and *fasn-1(D/E)* mutants had no effect on basal lipid droplet intensity as visualized by DHS-3::GFP (no stress; Fig. 5a). With challenges from ER, osmotic and ROS stress, *ced-3(-)* and *fasn-1(D/E)* mutants had a marked increase in lipid droplet intensity (Fig. 5a,b) but had no impact on the DHS-3::GFP protein levels (Extended Data Fig. 9a), suggesting increased accumulation of lipid droplets in *ced-3(-)* and *fasn-1(D/E)* mutants with stress treatments.

We next tested whether expressing FASN-CTF would impact the lipid droplet dynamics of *ced-3(-)* mutants. We found that the FASN-CTF was able to ameliorate the heightened lipid droplet accumulation in *ced-3(-)* mutants with ER (Fig. 5c), osmotic (Fig. 5d) and ROS stressors (Fig. 5e). When expressing FASN-CTF in wild-type animals, there were no differences in lipid droplets with ER, osmotic or ROS stressors (Extended Data Fig. 9b–d). This was expected, given that wild-type animals have functional CED-3 and FASN-1.

Given the ability of the FASN-CTF to promote cellular reductive capacity, we further tested how lipid droplet dynamics were affected by altering cellular redox conditions. We found that supplementing wild-type animals with the lipid aldehyde metabolite MDA enhances lipid droplet accumulation under ER stress (Fig. 5f), whereas supplementation with the antioxidant NAC ameliorated lipid droplet accumulation in *ced-3(-)* mutants (Fig. 5g). These findings indicate that expressing FASN-CTF had similar effects on lipid droplet dynamics as restoring cellular redox (Fig. 5h).

### FASN-CTF KR activity signals stress resolution

**Fig. 6:**
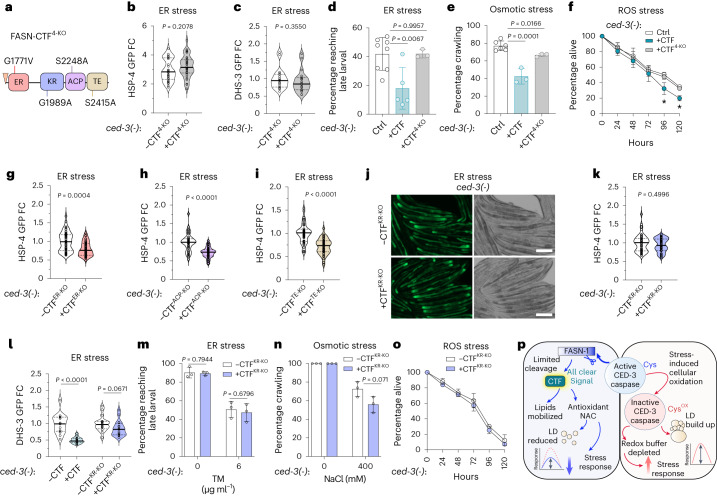
Impact of FASN-CTF enzymatic activity on pan-stress resolution and fitness. **a**, Diagram of FASN-CTF with four point mutations that inactivate the four conserved enzymatic domains (FASN-CTF^4-KO^). **b**, Induction of ER stress marker HSP-4p::GFP with expression of FASN-CTF^4-KO^ under ER stress in *ced-3(-)* mutant animals. *n* = 30 animals. **c**, Induction of LD marker DHS-3p::GFP with expression of FASN-CTF^4-KO^ under ER stress in *ced-3(-)* mutant animals. *n* = 18 animals. **d**–**f**, Artificially driving FASN-CTF expression during stress compromises survival. Developmental impact with either FASN-CTF or FASN-CTF^4-KO^ measured by developmental rate, motility and survival of *ced-3(-)* mutant animals under ER (**d**), osmotic (**e**) and ROS (**f**) stress conditions. *n* = 5 for CTF, *n* = 3 for CTF^4-KO^ and *n* = 8 for no CTF (Ctrl) biological replicates (**d**). Mean ± s.d. *n* = 3 for CTF and CTF^4-KO^ and *n* = 6 for no CTF biological replicates (**e**,**f**). Mean ± s.d. **g**–**i**, Quantification of HSP-4p::GFP with expression of FASN-CTF^ER-KO^ (enoyl reductase domain mutant) (**g**), FASN-CTF^ACP-KO^ (ACP domain mutant) (**h**) and FASN-CTF^TE-KO^ (TE domain mutant) (**i**) under ER stress in *ced-3(-)* mutant animals. *n* = 40 animals each. **j**,**k**, Image (**j**) and quantification (**k**) of HSP-4p::GFP with expression of FASN-CTF^KR-KO^ (KR domain mutant) under ER stress in *ced-3(-)* mutant animals. Scale bar, 200 μm. *n* = 40 animals each. **l**, Induction of LD marker DHS-3p::GFP with expression of FASN-CTF^KR-KO^ under ER stress in *ced-3(-)* mutant animals. *n* = 14 *ced-3(-)* with CTF, *n* = 15 *ced-3(-)* without CTF and *ced-3(-)* with or without CTF^4-KO^, *n* = 16 animals. **m**–**o**, Developmental impact with FASN-CTF^KR-KO^ measured by developmental rate, motility and survival of *ced-3(-)* mutant animals under ER (**m**), osmotic (**n**) and ROS (**o**) stress conditions. *n* = 3 biological replicates (**m**–**o**). Mean ± s.d. *P* values were obtained by two-tailed Mann–Whitney *U*-test (**b**,**c**,**g**–**i**,**k**,**l**) and unpaired, two-tailed *t*-test (**d**–**f**,**m**–**o**). **p**, Diagram illustrating the model of caspase CED-3 acting as stress sensor, generating FASN-CTF through cleavage of FASN to attenuate the stress response. Source data

Previous work identified point mutations that disrupt mammalian FASN enzymatic activity^1^. We introduced a transgene of FASN-CTF with point mutations of the highly conserved residues to inactivate each of the four domains in the FASN-CTF and referred to as FASN-CTF^4-KO^ (Fig. 6a). These residues were identified by homology to the human FASN (Extended Data Fig. 9e). FASN-CTF and FASN-CTF^4-KO^ were expressed at similar levels and were both responsive to auxin-induced degradation (Extended Data Fig. 9f). Analogous to FASN-CTF, expression of FASN-CTF^4-KO^ did not delay development under normal conditions, suggesting that it also does not impact endogenous FASN-1 function (Extended Data Fig. 9g). In contrast to FASN-CTF, expression of FASN-CTF^4-KO^ did not suppress the ER stress response as visualized by continued expression of HSP-4p::GFP (Fig. 6b and Extended Data Fig. 9h). Additionally, FASN-CTF^4-KO^ also did not ameliorate heightened lipid droplet accumulation (Fig. 6c and Extended Data Fig. 9i).

To distinguish whether the FASN-CTF functions more as an effector to mitigate stress or more as a signal to scale down stress responses, we assayed developmental progression under ER stress. We found that expression of FASN-CTF led to more stalled development for the *ced-3(-)* mutants in the presence of ER stress (+CTF; Fig. 6d). In contrast, FASN-CTF^4-KO^ behaved similar to *ced-3(-)* mutants (+CTF^4-KO^; Fig. 6d). Similarly, expression of FASN-CTF led to less motility under osmotic stress (Fig. 6e) and fewer survivors under ROS stress (Fig. 6f). Because expressing FASN-CTF decreased overall fitness of *ced-3(-)* mutants under diverse stress conditions, we concluded that the presence of FASN-CTF before stress mitigation compromises survival as it provides a premature stress-resolution signal.

**Extended Data Fig. 10:**
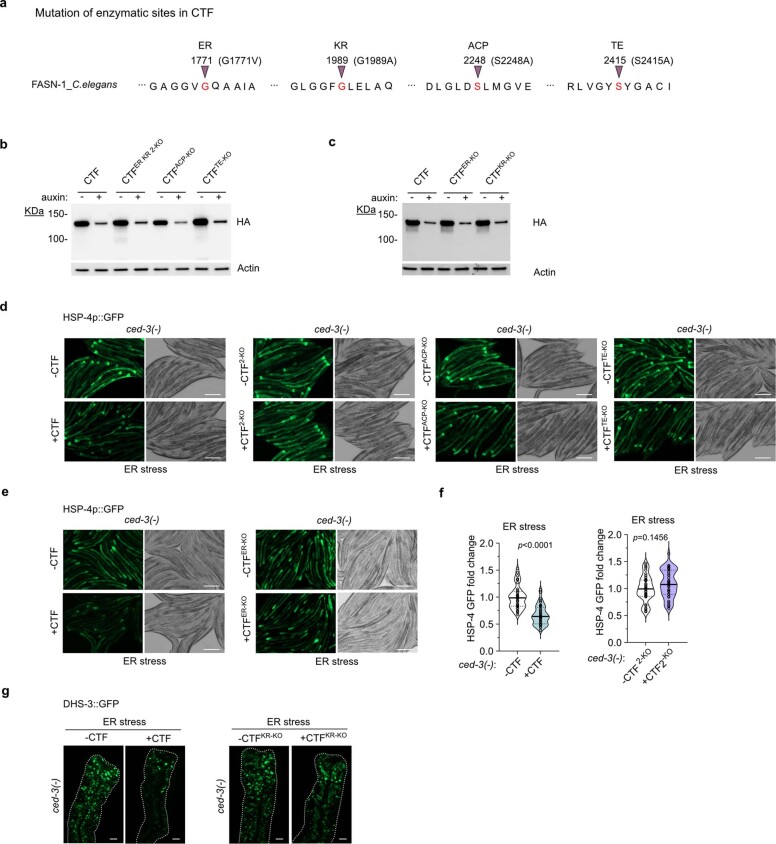
Characterization of FASN-CTF point mutations in individual enzymatic domain. **a**, local sequence with 4 point mutations that inactivate the four enzymatic domains. Single point mutations were generated to inactivate one site at a time. **b-c**, Western blots showing auxin-controlled expression of FASN-CTF point mutations including ER-KO (G1771V) KR-KO(G1989A), ACP-KO(S2248A), TE-KO(S2415A) and ER KR-2KO (G1771V, G1989A). Western blots were performed once to show no difference in expression. **d-e**, representative image of induction of ER stress marker HSP-4p::GFP with expression of FASN-CTF point mutations under ER stress in *ced-3(-)* mutant animals. Quantification shown in main Fig. 6g-i. **f**, Quantification of ER stress marker HSP-4p::GFP with expression of FASN-CTF point mutation ER KR-2KO (enoyl reductase and ketoacyl reductase domain mutants) and wild type FASN-CTF control under ER stress. n = 40 animals per condition. *p* values were calculated using Mann-Whitney, two-tailed test. **g**, representative image induction of lipid droplet marker DHS-3p::GFP with expression of FASN-CTF^KR-KO^ under ER stress in *ced-3(-)* mutant animals. Quantification shown in Fig. 6l. Scale bar, 200μm (**d**,**e**) and 10μm (**g**). Source data

These results suggest that catalytic activity of FASN-CTF is required for its function in attenuation of the stress response. To distinguish whether all four CTF domains or a specific domain were required, we generated single mutants to disrupt each of the enzymatic activities, including the enoyl reductase, KR, ACP and TE domains (Extended Data Fig. 10a) as these were mutated in the combined CTF 4-KO construct (Extended Data Fig. 10a). We found that each of the single mutants was expressed to similar extents as wild-type CTF and degraded in an auxin-dependent manner similar to wild-type CTF (Extended Data Fig. 10b,c). Elimination of the enoyl reductase, ACP or TE activities individually still allowed for suppression of HSP-4p::GFP induction (Fig. 6g–i and Extended Data Fig. 10d,e); however, disruption of either the enoyl reductase and KR domains jointly (2KO) or the KR domain alone (KR-KO) each abrogated the CTF suppression of HSP-4p::GFP induction (Fig. 6j,k and Extended Data Fig. 10d,f). Moreover, the KR domain single mutant failed to suppress accumulated lipid droplets (Fig. 6l and Extended Data Fig. 10g). In contrast to wild-type CTF, there was also no difference for *ced-3(-)* mutants expressing the CTF KR-KO domain single mutant when challenged with ER stress (Fig. 6m), osmotic stress (Fig. 6n) or ROS stress (Fig. 6o). Altogether, our findings suggest that the KR domain is critical for the FASN-CTF stress-suppression function.

In conclusion, we propose that FASN-CTF is generated by caspase cleavage in a redox-dependent manner. Under stress conditions, a more-oxidative cellular environment renders CED-3 caspase less active, leading to depleted redox buffer, lipid droplet accumulation and an increased stress response. Once stress is mitigated, a less-oxidative cellular environment reactivates caspase and generates FASN-CTF through proteolytic cleavage of FASN. FASN-CTF functions as an ‘all clear’ signal to initiate a stress response attenuation program. As an all clear signal, FASN-CTF is sufficient to promote lipid mobilization and reduce lipid droplet accumulation as well as downregulate stress response genes. As a result, physiological adaptations are re-programmed for a non-stress state to support growth and homeostasis (Fig. 6p).

## Discussion

In this study, we are proposing that FASN cleavage is a sensor that gauges a stress-free state. Under wild-type conditions, following restoration of redox balance, caspase is reactivated, allowing for cleavage and generation of FASN-CTF to downregulate the stress response (Fig. 6p). Thus, if FASN-CTF expression was forced without mitigating a stressor, this would provide an inappropriate downregulation of the stress response and would be detrimental to survival. Enhancing stress responsiveness improves survival during stressful states but is detrimental during stress-free conditions (chronic stress response). Conversely, diminishing a stress response prematurely would be detrimental without complete removal of the stressor (blunted stress response). Thus, organisms benefit from robust stress responses balanced with stress-sensing mechanisms.

Previous work analysing cell death factors, including *ced-3* caspase and *ced-4* Apaf, enhanced survival under stressful conditions through an unknown mechanism^4^. More recent work using a tamoxifen-induced ROS model demonstrated that the extent of lipid oxidative stress is modulated by the interplay of dietary fatty acids, host fatty acid metabolism and cell death pathways^29^. These findings suggest that innate adaptive mechanisms and host–diet interactions further alter the trajectory of stress responses. Moreover, that recent study also provided a scenario whereby, in response to an injury, some components, including oxidative status and cell death machinery, could themselves be entangled^29^. Additional recent findings show that ROS acting on dietary lipids compromises germ cell survival via ferroptosis and this can be alleviated by antioxidants^30^. Our present findings show an interplay of cellular redox and caspase cleavage of FASN in the determination of stress responsiveness.

Lipid biosynthesis itself has a notable function in activating stress responses. Compromised de novo fatty acid synthesis by mutation of *fasn-1* enhanced the expression of antimicrobial genes in *C.* *elegans*^23^ and oxidative stress response genes in yeast^31^; however, lipid biosynthesis by FASN-1 was shown to be required to initiate the mitochondria-to-cytosolic stress response, triggering HSF-1 and DVE-1 transcriptional programs^32^. Moreover, FASN is required for diet-induced inflammatory signaling^33^ as well as axonal regeneration following nerve injury^34^. These seemingly contradictory findings suggest a complex role for FASN in stress responses, which could be explained by the established lipid synthesis role as well as the signaling that we find.

Lipid droplets reflect an integrated readout of the stress response, protecting cells from excessive free lipids, sequestering toxic lipids, buffering ER stress by deposition of unfolded proteins and even serving as platforms for immune signaling^26^. Under stress, cells have a net increase in lipid droplets and their diminution is consistent with a diminished stress response. Previous studies have elegantly elucidated the regulation of lipid droplet formation and dynamics of turnover^28,35–40^, yet many questions remain unanswered, particularly with regard to the order of events and which steps represent full or reversible commitment in lipid droplet formation, release and dissolution. Further, despite considerable work showing the protective effects of lipid droplet formation, recent work has shown that lipid droplets can also be toxic when formed in the wrong context, such as in the nucleus of certain cells^41^. In this study, we reveal heightened lipid droplet accumulation in *ced-3(-)* and *fasn-1(D/E)* mutants consistent with a chronic stress response phenotype. Further, the enhanced accumulation of droplets along with other stress markers was ameliorated in *ced-3(-)* mutants by expression of FASN-CTF. Although we do not know the mechanism of lipid droplet reduction, it represents a readout of stress resolution.

Alterations in lipid accumulation activate SKN-1 Nrf, which itself is critical in lipid homeostasis^42^. Redox-sensitive transcriptional programs are essential to initiate an antioxidant response that mitigates oxidative challenges^43,44^. Thus, a reversible sensor acting at the post-translational level would reinforce the dynamic sensing of stress responses, allowing for rapid sequestration of energy stores. Limiting lipid peroxidation is of paramount importance for cell survival during stress^45^. Thus, it is noteworthy that the fatty acid-synthesizing enzyme FASN would also have a stress-sensing function, signaling the all clear after stressful encounters have been attenuated. Additionally, a shift in redox is a general feature of many stressful and pathogenic states, making redox restoration coupled with lipid droplet dynamics an effective adaptive response for stress mitigation. Moreover, accurately gauging a response proportional to the challenge without mounting an excessive response allows cellular energy supplies to be conserved. Redox signaling is complex, in that the redox factors can take on multiple roles^46,47^.

Efforts to map the cysteinome have revealed an array of oxidized cysteine species and complex roles of cysteine redox in signaling, gene expression, cellular growth and stress responses^48,49^. Depending on the oxidation state, multiple cysteine oxidation species are reversible^50,51^; however, some cysteine oxidation forms are irreversible^50,51^. Following redox resolution by mechanisms including SKN-1 Nrf transcriptional regulation^43,44,52^, NADPH oxidase signaling^53^ and subsequent sulfide production^54^, cysteine redox balance is collectively restored and the caspase active-site cysteine would be reactivated and allow for normal lipid droplet dynamics. Therefore, caspase makes for an intriguing sensor of oxidation state where mitigation of ROS could restore activity and allow resumed proteolytic activity. Future work will need to delineate whether redox restoration alone is sufficient or whether other factors in addition to redox are also required to restore caspase activity following stress responses in vivo. Either way, our findings show that caspase activity is altered by stress conditions.

Animals have evolved signaling mechanisms ranging from lipid-derived ligands to phosphorylation cascades to protein–protein interactions. In many cases, the signaling molecule or interaction responsible for these functions is present at minute levels, yet has potent roles as pro-development or stress-activating cues. Solving each of these elegant signaling mechanisms as multi-step cascades represented considerable effort with numerous studies. Our present findings reveal an unexpected role of FASN in detecting stress levels via a redox-sensitive, caspase-dependent proteolytic generation of the FASN-CTF. Of the four domains located in the CTF, we find that only the KR domain is essential for the stress-suppression function. The canonical activity of the KR enzyme family is to reduce acetoacetyl groups to hydroxyacyl groups in an NADP-dependent manner. Whether or not that is the relevant function in stress suppression will require significant future study. We find that generation of the FASN-CTF is a strong stress-resolution cue. Generation of the FASN-CTF can even be detrimental with forced expression of FASN-CTF without mitigation of the stressor. The duality of FASN as a biosynthetic enzyme and a generator of the FASN-CTF stress-resolution cue suggests that FASN may have a broader role as a signaling hub integrating basic metabolic demands and stress inputs.

## Methods

### Maintenance of *C.elegans* strains

All strains were maintained well fed at 20 °C for more than five generations before the experiments. Standard OP50 NGM plates were used unless otherwise indicated. RNAi clones were expressed in HT115 bacteria seeded on plates with 200 µg ml^−1^ ampicillin and 1 mM IPTG. For auxin treatments, 0.5 mM K-NAA auxin analogue (QJ-3105 Combi-Blocks) was used for each plate and animals were treated for the indicated times. Supplementary Table 1 lists all strains used in the study.

### Stress treatment and metabolite supplementation

TM (T7765, Sigma-Aldrich) was dissolved in dimethylsulfoxide (DMSO) as a 5 mg ml^−1^ stock for ER stress. Sodium chloride (S7653, Sigma-Aldrich) was dissolved in H_2_O as a 4 M stock for osmotic stress. Paraquat (856177, Sigma-Aldrich) was dissolved in H_2_O as a 1 M stock for ROS stress. For metabolite supplementation, 20 mg ml^−1^ stock solutions were made for each of the following metabolites: sodium palmitate (P9767, Sigma-Aldrich, in 1% NP-40), NAC (A7250, Sigma-Aldrich, in DMSO), l-carnosine (C9625, Sigma-Aldrich, in H_2_O), MDA (63287, Sigma-Aldrich, in H_2_O) and tetrabutylammonium chloride (86870, Sigma-Aldrich, in H_2_O).

To make NGM/TM agar plates with stressors or metabolite supplements, stock solution was diluted in H_2_O to a final volume of 400 µl as a working solution. The working solution was spread on an empty NGM agar plate surface to make the indicated concentration. Bacteria OP50 or HT115 was seeded once the surface had dried. An equal volume of solvent was used as a control. All plates were made fresh before the stress test.

For HSP-4p::GFP, NLP-29p::GFP and GCS-1p::GFP reporter induction, the same number of animals (~300 animals) were placed on each NGM plate treated with individual stressors. All animals were incubated at 20 °C overnight or at the indicated time points before imaging.

For metabolite supplementation, synchronized late-L3 animals were placed on an NGM plate with individual metabolite supplementation or solvent control for 6 h. TM (6 μg ml^−1^) was then added on each plate to induce ER stress overnight before imaging the next day.

### Phenotypical assays to evaluate stress treatment

Percent crawling with osmotic stress was scored by counting the number of motile animals. An animal was considered non-motile if it did not show forward or backward motion for 5 s. Percent alive with paraquat stress was scored by counting the number of worms alive at the indicated hour. An animal was considered alive if it showed head motion and pharyngeal pumping for 5 s. Percent developed to L4 or young adult with TM treatment was scored by spotting L1 synchronous animals to indicated plates and counting animals reaching the fourth larval stage or young adult after 2.5 d following treatment with TM. The s.d. and mean values are shown throughout.

### Growth rate assay

Five gravid young adults were allowed to lay eggs on each plate synchronously with or without stressors and removed after 3 h. Animals were staged at 62–68 h when more than 80% control animals with no stress treatment reached young adulthood. Before testing, animals were maintained under stress-free conditions at 20 °C for multiple generations, including no starvation and no obvious contamination.

### RNAi screen

RNAi screening was performed on RNAi NGM agar plates. Animals were placed on RNAi plates and allowed to grow for 6 h. Plates were then treated with 400 µl diluted DMSO or TM (6 μg ml^−1^) and allowed to soak in for 30 min. The induction of HSP-4p::GFP was imaged 22 h later.

### Generation of CRISPR-Cas9 mutations

Dual HA-AID-tagged FASN-1 (HA::AID::GSGTGS::FASN-1::GSGTGS::AID::HA), caspase cleavage-resistant FASN-1 (D1593E) point mutation and CED-3::HA were generated using short-range HDR with *dpy-10* co-CRISPR method in the endogenous genomic locus. An extra single-copy transgene of FASN-NTF (HA::AID::GSGTGS::FASN-1 (aa2-aa1593)), FASN-NTF^Δlinker^ (HA::AID::GSGTGS::FASN-1 (aa2-aa1138)), FASN-1-CTF (FASN-1 (aa1594 to aa2613)::GSGTGS::AID::HA) and FASN-1-CTF^4-KO^ was inserted into Mos1 transposon site LGIII Mos1_oxTi444 or LGI Mos1_ttTi4348 using the SEC CRISPR-Cas9 method.

For cell autonomy testing, a body-wall muscle promoter (myo-3) and intestine promoter (ges-1) were used to drive the expression of FASN-CTF as extra single-copy transgenes. To generate these tissue-specific-expressing FASN-CTF strains, CRISPR mutagenesis was used to insert one extra copy of each transgene in the LGIII Mos1_oxTi444 transposon site and crossing each of them into a ced-3 null mutant expressing the HSP-4p::GFP reporter, respectively. Strain’s names, rescue templates and single-guide RNAs are shown in Supplementary Tables 1–3.

### Imaging

For imaging analysis, animals were immobilized with 5% sodium azide (UN3287 ICCA) at the indicated time point. For HSP-4p::GFP, NLP-29p::GFP and GCS-1p::GFP stress reporter inductions, images were acquired using an Axiozoom microscope (Zeiss). For DHS-3::GFP LD visualization, images were acquired using a Nomarski microscope (Zeiss running Zen v.2.5) with ApoTome. All fluorescence quantifications were processed using Fiji (ImageJ v.1.54f). For quantification, the animal boundary was defined using bright-field imaging and the mean pixel intensity was calculated using a fluorescent image taken with the same field of view. Quantification was presented as FC to WT animals at 0 h for a given treatment. For confocal microscopy of Grx1-GFP2 strains, images were acquired using a Zeiss LSM900 equipped with Airyscan 2, GaAsP PMT detectors and 405/488/561/640-nm lasers running Zen v.3.4. Specifically, excitation by a 405-nm laser and a 488-nm laser was performed in tandem to determine the relative fractions of oxidized and reduced Grx1-roGFP2, respectively. Emissions were detected by a GaAsP PMT with no filters.

### In vitro cleavage assay

Caspase cleavage reactions were performed as previously reported^55^. Purified human caspase-2 (R&D Systems 702-C2-010/CF), caspase-3 (R&D Systems 707-C3-010/CF) and caspase-8 (R&D Systems 705-C8-010/CF) were incubated with ^35^S-labelled FASN fragments at 30 °C temperature overnight. ^35^S-labelled substrates were synthesized fresh before cleavage reactions using a TNT system (Promega). Cleavage reactions were stopped by adding sample buffer and heating to 85 °C. Samples were resolved by SDS–PAGE on 4–16% gradient gels and dried before imaging.

### Western blot

Animals were washed off plates using M9 and snap frozen with liquid nitrogen. Pellets were sonicated in lysis buffer containing 10 mM Tris, pH 7.4, 1 mM EDTA, 150 mM NaCl, 0.5 % NP-40, Halt Protease and Phosphatase Inhibitor Cocktails (Fisher Scientific, PI78440) on ice. Approximately 5 μg of total protein was loaded per well and resolved on 4–20% gradient acrylamide gels. Anti-HA antibody from rabbit (Cell Signaling Technology, 3724S) was used at 1:1,000 dilution. Anti-actin (Bio-Rad, 12004163) antibody was used at 1:4,000 dilution for loading control.

### Immunoprecipitation of caspase

An HA tag was inserted at the C terminus of CED-3 using CRISPR mutagenesis. Because CED-3 expression is extremely low, HA immunoprecipitation (IP) was undertaken to observe the expression and processing of CED-3. Synchronized L4 was treated with 4 μg ml^−1^ TM for 18 h and 100 μl worm pellet was collected and snap frozen in liquid N_2_ for IP. The worm pellet was lysed using sonication and the protein concentration was determined using a BCA assay. IP was performed using HA tag (C29F4) rabbit monoclonal antibody (Cell Signaling Technology, 11846S) at 1:200 dilution for 4 h at 4 °C. Western blot was performed using mouse monoclonal antibody (Cell Signaling Technology, 2367S). Actin (Bio-Rad, 12004163) was used as a loading control of the input.

### Free fatty acid isotopologues

Synchronized L4 animals were collected and incubated with PBS in 50% deuterium-labelled water for 20 h with rotation. UV-killed bacteria were used as a food source to limit additional metabolism. Animals were collected in M9 and the worm pellet was snap frozen in liquid N_2_. Worms were lysed in 1 ml ice-cold 50% methanol with sonication and 1% BHT was added to prevent lipid oxidation. Free fatty acids were extracted by mixing 0.5 ml chloroform to each sample to reach a methanol:water:chloroform ratio of 1:1:1 (*v*/*v*/*v*). Samples were then spun at 1,000*g* for 5 min and the organic phases (lower) were collected and dried under air flow. Then, 100 µl 1 N HCl and 200 µl toluene were sequentially added with vortexing to each sample. After a brief spin, the toluene solution (top layer) was decanted into a fresh Eppendorf tube and evaporated to dryness. Then, 100 µl of TBDMS solution (Sigma) was added to each sample with vortexing and heated to 70 °C for 1 h for fatty acid derivatization. Samples were then transferred to gas chromatograph vials and run on an Agilent 6970 gas chromatograph networked to an Agilent 5973 Mass Selective Detector. Fragment ion *m*/*z* 313–329 was used to monitor enrichment in palmitate.

### Metabolomics

Synchronized very young adults before egg laying were collected using M9 and washed 2× with Milli-Q water. The worm pellet was about 100 mg wet weight. Then, 85% methanol (400 μl) was added with 0.1 mm zirconia beads (RPI 9833) for each extraction using a FastPrep-24 sample preparation system (MP Biomedicals). The supernatant was transferred to a clean tube after 1 min of 16,000*g* centrifugation. A BCA assay was used to determine the protein concentration. Samples were dried with a speed vacuum (Eppendorf) and resuspended in acetonitrile/water for metabolomics analysis.

Untargeted metabolomics was performed by Creative Proteomics. A targeted metabolomics screen was conducted by the University of Texas Southwestern (UTSW) CRI metabolomics core. A *t*-test was used to evaluate the significance in metabolite change between two sets of samples. The MetaboAnalyst (www.metaboanalyst.ca) pathway enrichment module was used for pathway analysis using the SMPDB. The ranking of the enrichment pathway was based on both *P* value and the percent of metabolites in a given pathway represented in the metabolomics panel. GraphPad was used for PCA and eigenvalue calculations.

### Seahorse measurement of oxygen consumption rate

Plates of synchronized adult worms were collected by washing with 1 ml M9 buffer. The worms were allowed to settle to the bottom by gravity and then rinsed three times with fresh M9 buffer to remove bacteria. The worms were then plated on sterile NGM plates in a 300-μl drop of M9 buffer. The worms were allowed to crawl out of the drop and then placed in a seahorse assay plate in 200 μl M9 buffer. Each well contained 40 adult worms. The worms were allowed to acclimate to the buffer for 30 min and then OCR was measured three times using a seahorse XFp. The amount of mitochondrial-specific respiration was determined by injecting sodium azide (40 mM final concentration) and measuring the residual OCR. Each data point represents the average of three OCR measurements. Each experiment contained three independent biological replicates.

### mRNA-seq

Synchronous young-adult-stage animals were collected in TRIzol reagent (Invitrogen, 15596-026). Total RNA was extracted and DNase treated. Three independent biological replicates of each treatment and genotype were collected. Libraries were generated, sequenced and analysed for differential gene expression by the UTSW McDermott Center Next-Generation Sequencing Core. For messenger RNA-seq, single-end 76-bp-read-length fastq files were checked for quality using fastqc (v.0.11.2) and fastq_screen (v.0.4.4). Reads were quality trimmed using fastq-mcf (ea-utils, v.1.1.2-806). Trimmed fastq files were mapped to the *C.* *elegans* genome using STAR (v.2.5.3a). Duplicate alignments were removed using Picard Tools (v.2.10). Read counts were generated using featureCounts (subread) for coding genes from iGenomes (RefSeq). Normalized read counts were generated using edgeR. WormBase gene set enrichment analysis tools were used for Gene Ontology analyses.

### DCP-Bio1 labelling and proteomic analysis

Primary cOBs were isolated as described previously^56^. cOBs were cultured with or without 250 μM H_2_O_2_ for 24 h before DCP-Bio1 labelling. DCP-Bio1 selectively reacts with cysteine sulfenic acid thus detecting oxidation products. Cells were scraped, lysed and reacted with the cysteine sulfenic acid probe, 3-(2,4-dioxocyclohexyl) propyl appended to biotin (DCP-Bio1) for 1.5 h in a modified lysis buffer containing 50 mM Tris-HCl (pH 7.5), 100 mM NaCl, 0.1% SDS, 0.5% sodium deoxycholate, 0.5% NP-40, 0.5% Triton X-100, 50 mM NaF, 1 mM phenylmethylsulfonyl fluoride, 1 mM DCP-Bio1, 100 µM diethylenetriamine pentaacetic acid, 10 mM *N*-ethylmaleimide, 10 mM iodoacetamide, 200 U ml^−1^ catalase and protease inhibitor cocktail. After centrifugation at 9,600*g* for 10 min, unreacted DCP-Bio1 in the supernatant was removed using Bio-Rad P6-Spin columns. DCP-Bio1-labelled proteins were immunoprecipitated using a Pierce Classic Magnetic IP kit and purified with 10% SDS–PAGE gel. Proteomic analysis of gel slices was performed by the University of Texas Southwestern Proteomics Core.

### Mouse fasting assay

Mice used in this study were all WT males on C57BL/6J background. Mice were housed in a specific pathogen-free facility with a 12-h light–dark cycle and fed with standard rodent chow ad libitum. The housing temperature range was 68–79 °F and humidity was 30–70%. During fasting, each mouse was individually caged with free access to water. Body weight was monitored every 24 h. Mice were killed at the end of 36 h of fasting. The liver was collected and snap frozen in liquid nitrogen. All animal use was approved by the Institutional Animal Care and Use Committee at the UTSW Medical Center.

### Statistics

Statistical analyses and *P* values are provided in figures, legends, Supplementary Data 1–3 and Source Data.

### Reporting summary

Further information on research design is available in the Nature Portfolio Reporting Summary linked to this article.

## Supplementary information

Reporting Summary

Supplementary Table 1 *C.* *elegans* strains used in this study.

Supplementary Table 2 Plasmids used in this study.

Supplementary Table 3 Oligonucleotides used in this study.

Supplementary Data 1 Untargeted metabolomics for wild-type, *ced-3(-)* and *fasn-1(D/E)* with no stress, ER, osmotic and ROS stress.

Supplementary Data 2 mRNA-seq differential expression of *ced-3(-)* with or without FASN-CTF expression under ER stress.

Supplementary Data 3 Targeted metabolomics for wild-type and *ced-3(-)* with or without FASN-CTF expression under ER stress.

## Source data

Source Data Fig. 1 Statistical Source Data.

Source Data Fig. 2 Unprocessed gels.

Source Data Fig. 2 Statistical Source Data.

Source Data Fig. 3 Unprocessed western blots.

Source Data Fig. 3 Statistical Source Data.

Source Data Fig. 4 Unprocessed western blots.

Source Data Fig. 4 Statistical Source Data.

Source Data Fig. 5 Statistical Source Data.

Source Data Fig. 6 Statistical Source Data.

Source Data Extended Data Fig. 1 Statistical Source Data.

Source Data Extended Data Fig. 2 Statistical Source Data.

Source Data Extended Data Fig. 3 Unprocessed western blots.

Source Data Extended Data Fig. 3 Statistical Source Data.

Source Data Extended Data Fig. 4 Unprocessed western blots.

Source Data Extended Data Fig. 4 Statistical Source Data.

Source Data Extended Data Fig. 5 Statistical Source Data.

Source Data Extended Data Fig. 6 Statistical Source Data.

Source Data Extended Data Fig. 7 Statistical Source Data.

Source Data Extended Data Fig. 8 Unprocessed western blots.

Source Data Extended Data Fig. 8 Statistical Source Data.

Source Data Extended Data Fig. 9 Unprocessed western blots.

Source Data Extended Data Fig. 9 Statistical Source Data.

Source Data Extended Data Fig. 10 Unprocessed western blots.

Source Data Extended Data Fig. 10 Statistical Source Data.

## Data Availability

The mRNA-seq data generated in this study have been deposited in the Gene Expression Omnibus database under accession code GSE226048. The remaining data generated in this study are provided in the Source Data file. Source data are provided with this paper.
