# Supplementary figures and images for: Proteolytic activation of fatty acid synthase signals pan-stress resolution
Source: Nat Metab. 2024 Jan 2;6(1):113–26. doi: 10.1038/s42255-023-00939-z (PMC10822777; doi:10.1038/s42255-023-00939-z)

Fig 2c

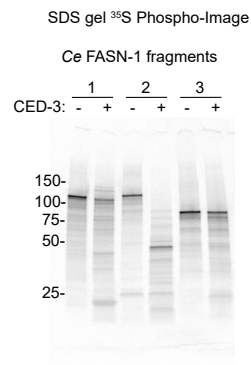

Fig 2d

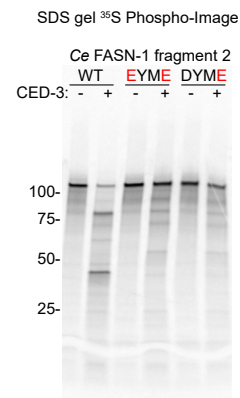

Fig 2e

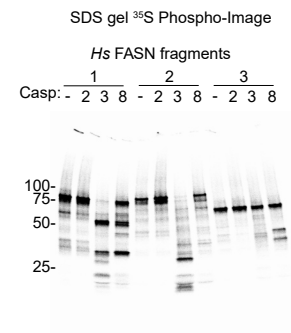

Supplement: Supplementary file 9 — Unprocessed gels. [file 42255_2023_939_MOESM9_ESM.pdf]

Fig.3i

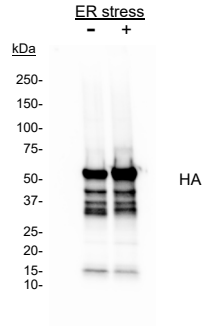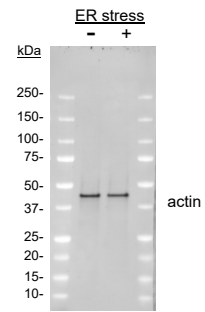

Fig.3j

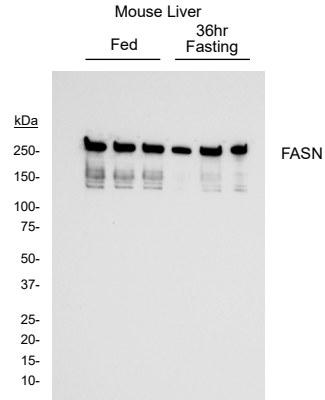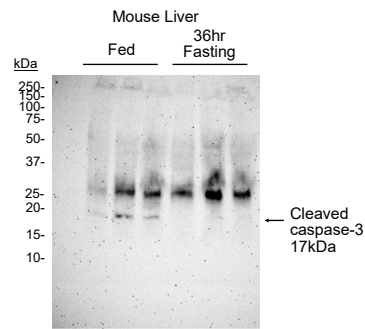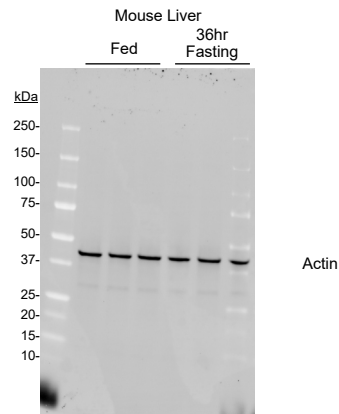

Supplement: Supplementary file 11 — Unprocessed western blots. [file 42255_2023_939_MOESM11_ESM.pdf]

Fig.4a

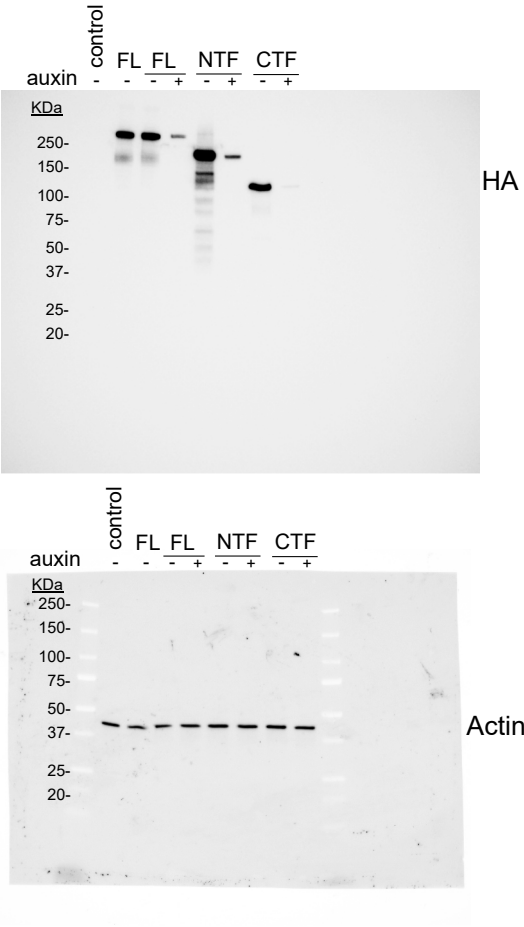

Supplement: Supplementary file 13 — Unprocessed western blots. [file 42255_2023_939_MOESM13_ESM.pdf]

Extended Fig.3d

|                       |   |   |   |    |    |    |
|-----------------------|---|---|---|----|----|----|
| TIR-1 E3 Ligase       | - | - | + | +  | +  | +  |
| FASN-1 <sup>AID</sup> | - | + | + | +  | +  | +  |
| auxin                 | - | - | + | +  | +  | +  |
| time(h)               | 0 | 0 | 0 | 16 | 24 | 32 |

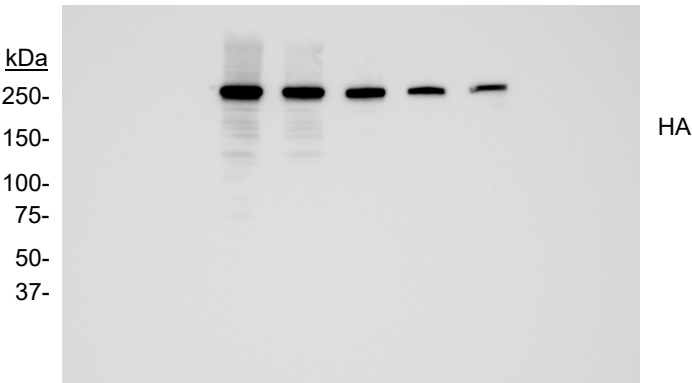

|                       |   |   |   |    |    |    |
|-----------------------|---|---|---|----|----|----|
| TIR-1 E3 Ligase       | - | - | + | +  | +  | +  |
| FASN-1 <sup>AID</sup> | - | + | + | +  | +  | +  |
| auxin                 | - | - | + | +  | +  | +  |
| time(h)               | 0 | 0 | 0 | 16 | 24 | 32 |

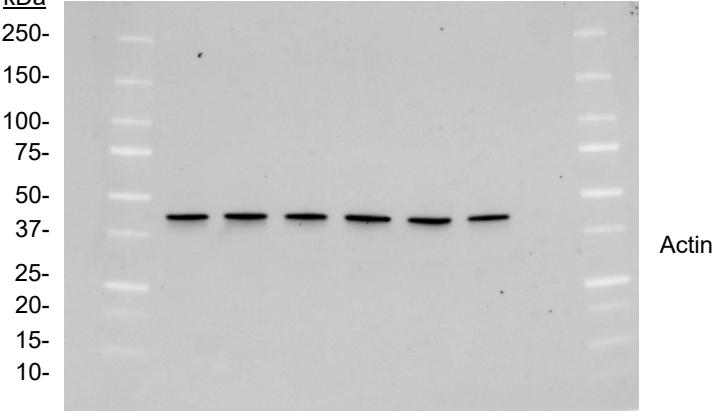

Supplement: Supplementary file 19 — Unprocessed western blots. [file 42255_2023_939_MOESM19_ESM.pdf]

Extended Fig.4h

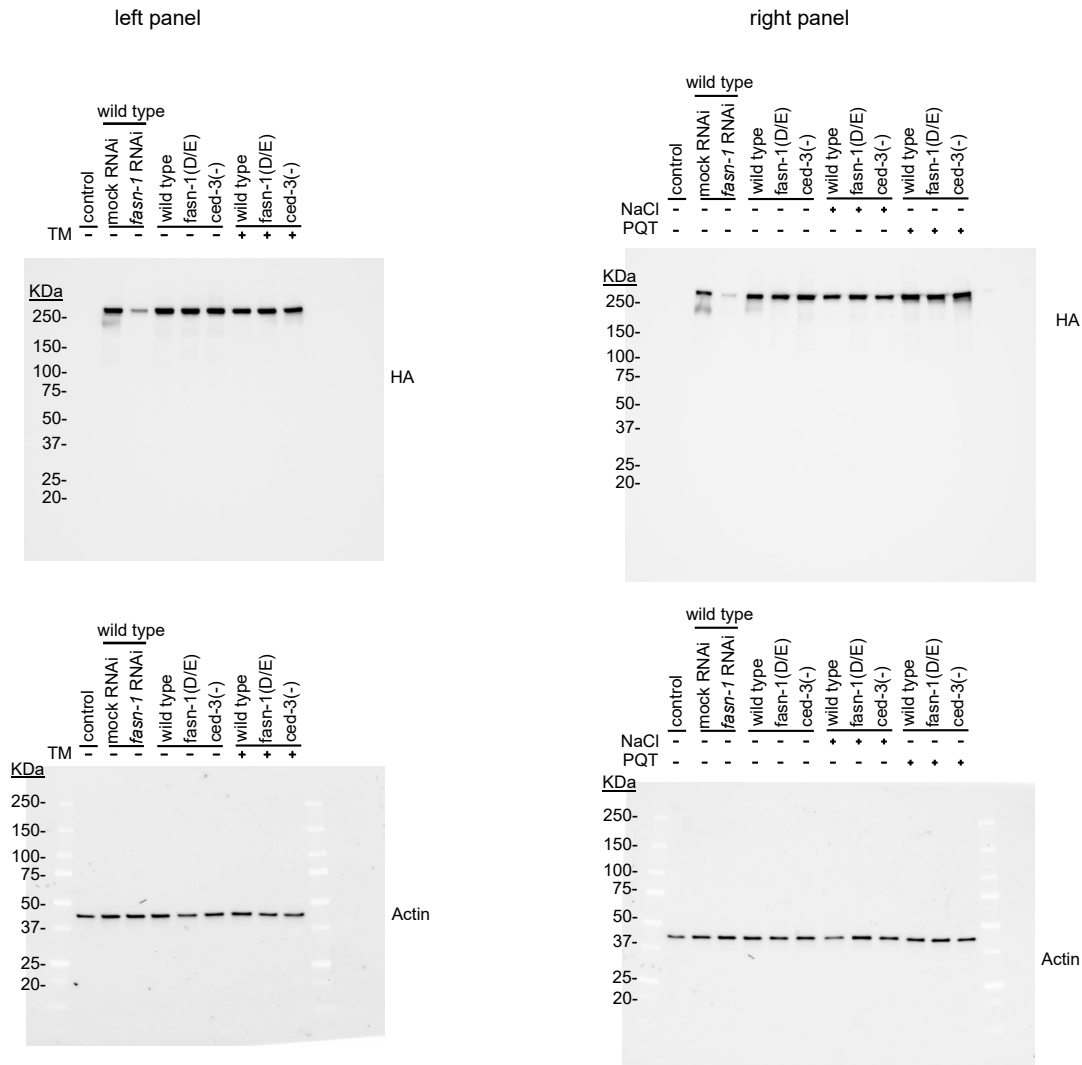

Supplement: Supplementary file 21 — Unprocessed western blots. [file 42255_2023_939_MOESM21_ESM.pdf]

Extended Fig.8b

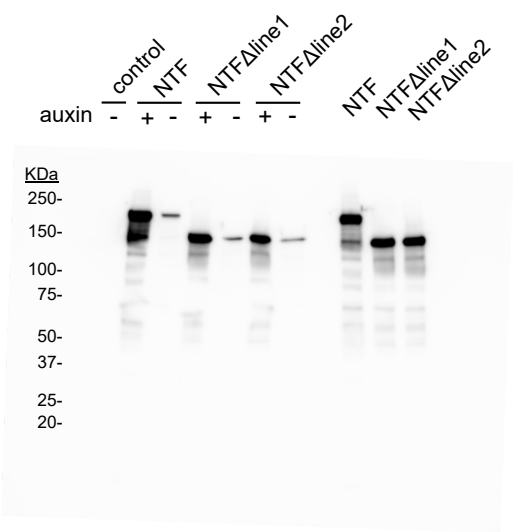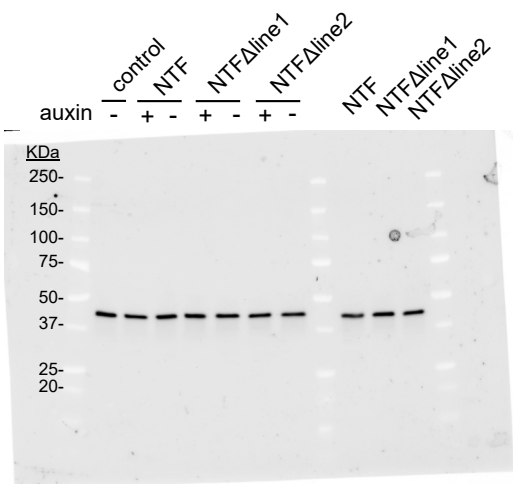

Extended Fig.8c

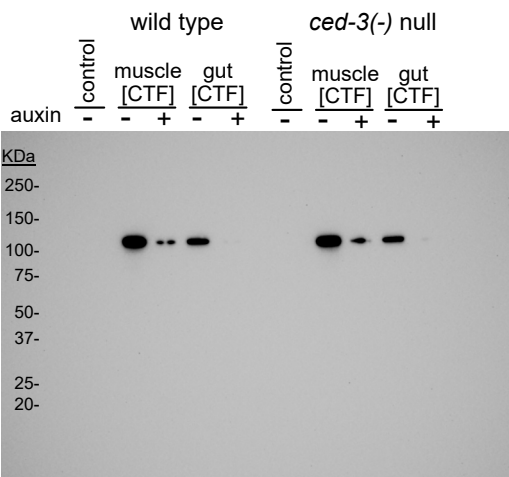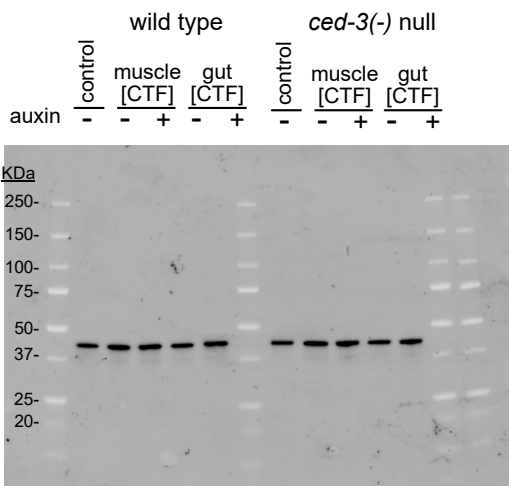

Supplement: Supplementary file 26 — Unprocessed western blots. [file 42255_2023_939_MOESM26_ESM.pdf]

Extended Fig.9a

Left panel

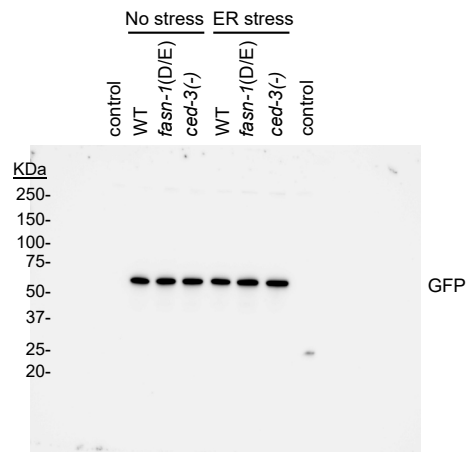

Right panel

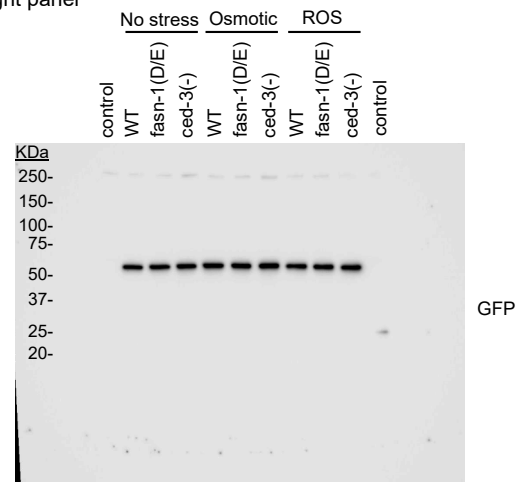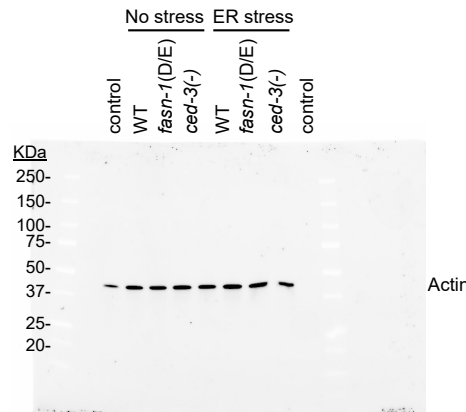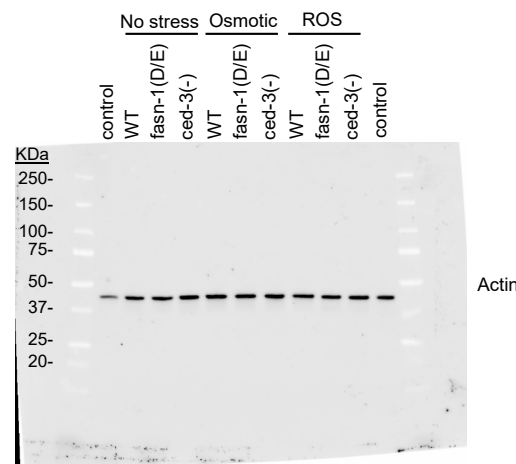

Extended Fig.9f

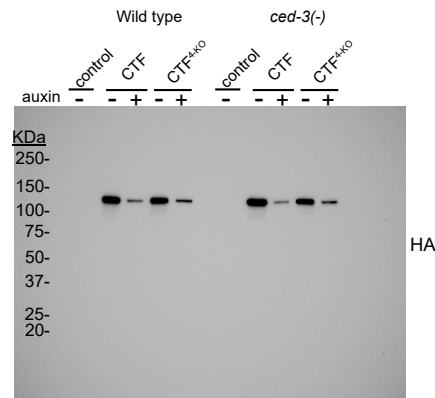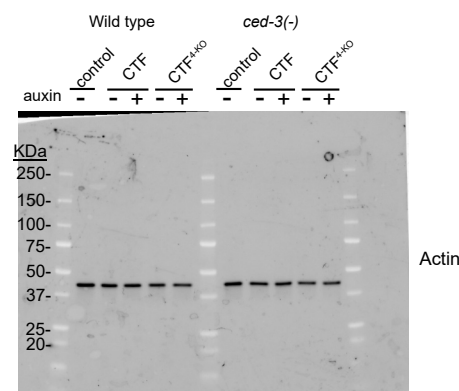

Supplement: Supplementary file 28 — Unprocessed western blots. [file 42255_2023_939_MOESM28_ESM.pdf]

Extended Fig.10b

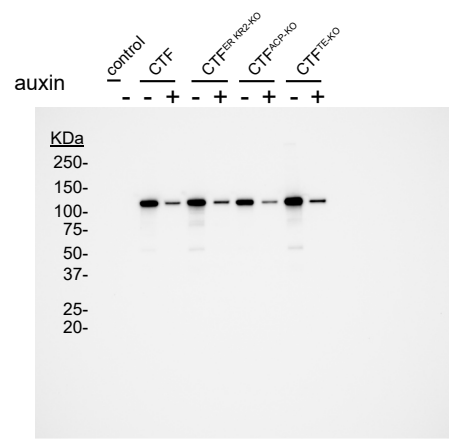

Extended Fig.10c

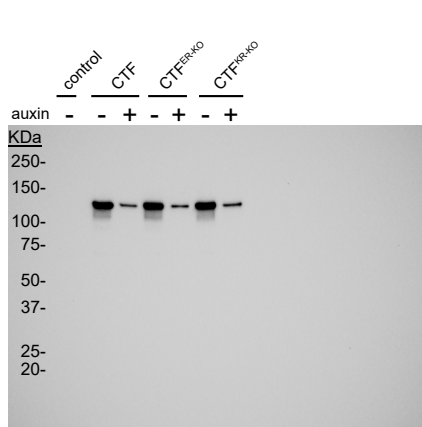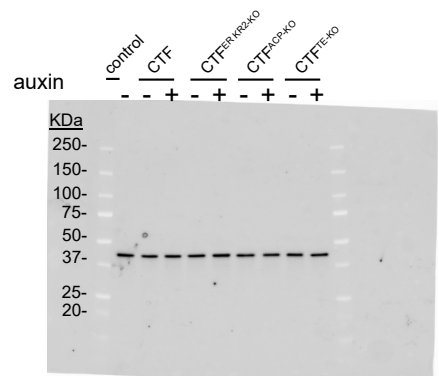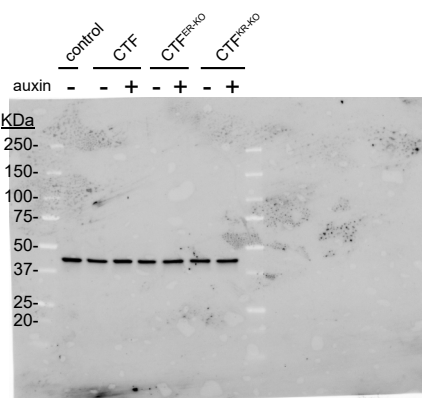

Supplement: Supplementary file 30 — Unprocessed western blots. [file 42255_2023_939_MOESM30_ESM.pdf]
